# CONSORT-SPI 2018 Explanation and Elaboration: guidance for reporting social and psychological intervention trials

**DOI:** 10.1186/s13063-018-2735-z

**Published:** 2018-07-31

**Authors:** Sean Grant, Evan Mayo-Wilson, Paul Montgomery, Geraldine Macdonald, Susan Michie, Sally Hopewell, David Moher, , on behalf of the CONSORT-SPI Group, J. Lawrence Aber, Doug Altman, Kamaldeep Bhui, Andrew Booth, David Clark, Peter Craig, Manuel Eisner, Mark W. Fraser, Frances Gardner, Sean Grant, Larry Hedges, Steve Hollon, Sally Hopewell, Robert Kaplan, Peter Kaufmann, Spyros Konstantopoulos, Geraldine Macdonald, Evan Mayo-Wilson, Kenneth McLeroy, Susan Michie, Brian Mittman, David Moher, Paul Montgomery, Arthur Nezu, Lawrence Sherman, Edmund Sonuga-Barke, James Thomas, Gary VandenBos, Elizabeth Waters, Robert West, Joanne Yaffe

**Affiliations:** 1 Behavioral & Policy Sciences, RAND Corporation, 1776 Main Street, Santa Monica, CA 90407-2138 USA; 2 Department of Epidemiology, Johns Hopkins University Bloomberg School of Public Health, 615 North Wolfe Street, E6036, Baltimore, MD 21205 USA; 3 School of Social Policy, University of Birmingham, Edgbaston, Birmingham, B15 2TT UK; 4 School for Policy Studies, 8 Priory Road, Bristol, BS8 1TZ UK; 5 Department of Clinical, Educational and Health Psychology, Centre for Behaviour Change, University College London, London, WC1E 7HB UK; 6 Oxford Clinical Trials Research Unit, Nuffield Department of Orthopaedics, Rheumatology, and Musculoskeletal Sciences, University of Oxford, Botnar Research Centre, Windmill Road, Oxford, OX3 7LD UK; 7 Centre for Journalology, Clinical Epidemiology Program, Ottawa Hospital Research Institute, Ottawa, ON K1H 8L6 Canada

**Keywords:** CONSORT, Randomised controlled trial, Reporting guideline, Reporting standards, Transparency

## Abstract

**Background:**

The CONSORT (Consolidated Standards of Reporting Trials) Statement was developed to help biomedical researchers report randomised controlled trials (RCTs) transparently. We have developed an extension to the CONSORT 2010 Statement for social and psychological interventions (CONSORT-SPI 2018) to help behavioural and social scientists report these studies transparently.

**Methods:**

Following a systematic review of existing reporting guidelines, we conducted an online Delphi process to prioritise the list of potential items for the CONSORT-SPI 2018 checklist identified from the systematic review. Of 384 international participants, 321 (84%) participated in both rating rounds. We then held a consensus meeting of 31 scientists, journal editors, and research funders (March 2014) to finalise the content of the CONSORT-SPI 2018 checklist and flow diagram.

**Results:**

CONSORT-SPI 2018 extends 9 items (14 including sub-items) from the CONSORT 2010 checklist, adds a new item (with 3 sub-items) related to stakeholder involvement in trials, and modifies the CONSORT 2010 flow diagram. This Explanation and Elaboration (E&E) document is a user manual to enhance understanding of CONSORT-SPI 2018. It discusses the meaning and rationale for each checklist item and provides examples of complete and transparent reporting.

**Conclusions:**

The CONSORT-SPI 2018 Extension, this E&E document, and the CONSORT website (www.consort-statement.org) are helpful resources for improving the reporting of social and psychological intervention RCTs.

**Electronic supplementary material:**

The online version of this article (10.1186/s13063-018-2735-z) contains supplementary material, which is available to authorized users.

## Background

### CONSORT-SPI 2018 explanation and elaboration

The CONSORT (Consolidated Standards of Reporting Trials) Statement was developed to help authors report randomised controlled trials (RCTs) [1]. It has improved the quality of reports in medicine [2–5], and has been officially endorsed by over 600 journals and prominent editorial groups [6]. A smaller number of journals have implemented CONSORT—particularly its extension statements—as a requirement for the manuscript submission, peer-review, and editorial decision-making process [6, 7]. There are extensions of the CONSORT Statement (http://www.consort-statement.org/extensions) for specific trial designs [8–11], types of data (e.g. patient-reported outcomes, harms, and information in abstracts) [12–14], and interventions [15–17].

Several reviews have shown that RCTs of social and psychological interventions are often not reported with sufficient accuracy, comprehensiveness, and transparency to replicate these studies, assess their quality, and understand for whom and under what circumstances the evaluated intervention should be delivered [18–22]. Moreover, behavioural and social scientists may be prevented from reproducing or synthesising previous studies because trial protocols, outcome data, and the materials required to implement social and psychological interventions are often not shared [23–28]. These inefficiencies contribute to suboptimal dissemination of effective interventions [29, 30], overestimation of intervention efficacy [31], and research waste [29]. Transparent and detailed reporting of social and psychological intervention RCTs is needed to minimise reporting biases and to maximise the credibility and utility of this research evidence [32, 33].

We developed an extension of the CONSORT 2010 Statement for Social and Psychological Interventions (CONSORT-SPI 2018) [34]. To delineate the scope of CONSORT-SPI 2018, we defined interventions by their mechanisms of action [35, 36]. We define social and psychological interventions as actions intended to modify processes and systems that are social and psychological in nature (such as cognitions, emotions, behaviours, norms, relationships, and environments) and are hypothesised to influence outcomes of interest [37, 38]. Social and psychological interventions may be offered to individuals who request them or as a result of policy, may operate at different levels (e.g., individual, group, place), and are usually “complex” [19]. CONSORT-SPI 2018 is designed primarily for reports of RCTs, though some parts of this guidance may be useful for researchers conducting other types of clinical trials [39] or who are interested in developing and evaluating complex interventions [40]. In addition, although terms in this report are most appropriate for parallel-group trials, the guidance is designed to apply to other designs (e.g. stepped wedge and N of 1).

## Methods

We previously reported the methods used to develop CONSORT-SPI 2018 [41]. In summary, we first conducted systematic reviews of existing guidance and quality of trial reporting [18]. Second, we conducted an international online Delphi process between September 2013 and February 2014 to prioritise the list of potential items for the CONSORT-SPI 2018 checklist and flow diagram that were identified in the systematic review. Survey items can be accessed at the project’s ReShare site: 10.5255/UKDA-SN-851981. Third, we held a consensus meeting to finalise the content of the checklist and flow diagram. The meeting was held in March 2014 and comprised 31 scientists, journal editors, and research funders. A writing group drafted CONSORT-SPI 2018, and consensus group participants provided feedback and agreed to the final manuscript for the CONSORT-SPI 2018 Extension Statement [42], and this Explanation and Elaboration (E&E) document (Additional file 1: Table S1).

**Fig. 1:**
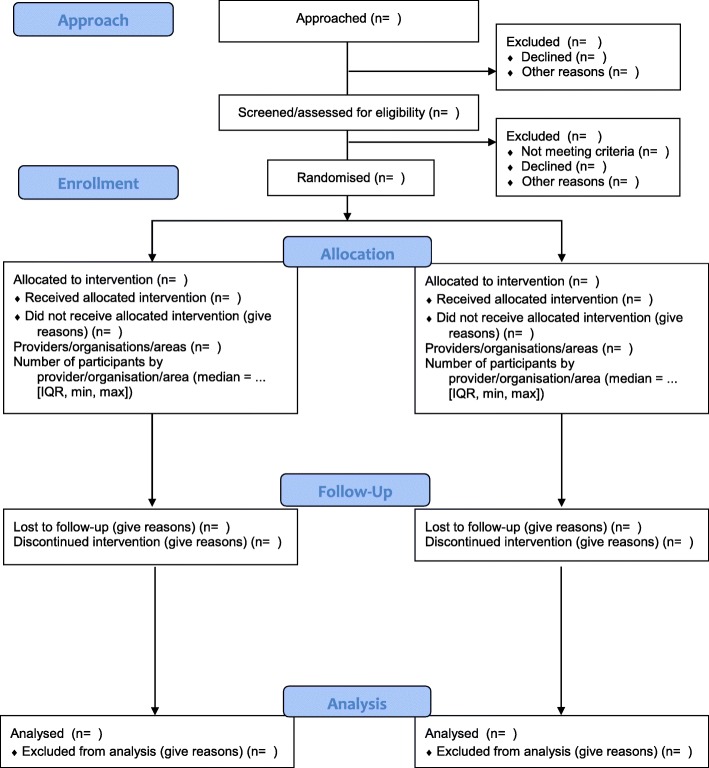
The CONSORT-SPI 2018 flow diagram

The CONSORT-SPI 2018 checklist extends 9 of the 25 items (incorporating 14 sub-items) found in CONSORT 2010 (Table 1; new items are in the ‘CONSORT-SPI 2018’ column) and includes a modified flow diagram. Participants also voted to add a new item about stakeholder involvement, and they recommended modifications to existing CONSORT 2010 checklist items (Table 2). This E&E document briefly summarises the content from the CONSORT 2010 E&E document for each CONSORT 2010 item [43], tailored to a behavioural and social science audience, and it provides an explanation and elaboration for the new items in CONSORT-SPI 2018. Specifically, for each item from CONSORT 2010 and CONSORT-SPI 2018, this E&E provides the rationale for the checklist item and examples of reporting for a behavioural and social science audience (Additional file 2: Table S2). Throughout this article, we use the term ‘participants’ to mean ‘participating units’ targeted by interventions, which might be individuals, groups, or places (i.e. settings or locations) [44]. While we include a brief statement about each item in the CONSORT 2010 checklist, readers can find additional information about these items on the CONSORT website (www.consort-statement.org) and the CONSORT 2010 E&E [43].

**Table 1:** The CONSORT-SPI 2018 checklist

Section	Item #	CONSORT 2010	CONSORT-SPI 2018
Title and abstract	1a	Identification as a randomised trial in the title^**§**^	
	1b	Structured summary of trial design, methods, results, and conclusions (for specific guidance, see CONSORT for Abstracts)^**§**^	Refer to CONSORT extension for social and psychological intervention trial abstracts
Introduction			
Background and objectives	2a	Scientific background and explanation of rationale^**§**^	
	2b	Specific objectives or hypotheses^**§**^	If pre-specified, how the intervention was hypothesised to work
Methods			
Trial design	3a	Description of trial design (such as parallel, factorial) including allocation ratio^**§**^	If the unit of random assignment is not the individual, please refer to CONSORT for Cluster Randomised Trials [8]
	3b	Important changes to methods after trial commencement (such as eligibility criteria), with reasons	
Participants	4a	Eligibility criteria for participants^**§**^	When applicable, eligibility criteria for settings and those delivering the interventions
	4b	Settings and locations where the data were collected	
Interventions	5	The interventions for each group with sufficient details to allow replication, including how and when they were actually administered^**§**^	
	5a		Extent to which interventions were actually delivered by providers and taken up by participants as planned
	5b		Where other informational materials about delivering the intervention can be accessed
	5c		When applicable, how intervention providers were assigned to each group
Outcomes	6a	Completely defined pre-specified outcomes, including how and when they were assessed^**§**^	
	6b	Any changes to trial outcomes after the trial commenced, with reasons	
Sample size	7a	How sample size was determined^**§**^	
	7b	When applicable, explanation of any interim analyses and stopping guidelines	
Randomisation			
Sequence generation	8a	Method used to generate the random allocation sequence	
	8b	Type of randomisation and details of any restriction (such as blocking and block size)^**§**^	
Allocation concealment mechanism	9	Mechanism used to implement the random allocation sequence, describing any steps taken to conceal the sequence until interventions were assigned^**§**^	
Implementation	10	Who generated the random allocation sequence, who enrolled participants, and who assigned participants to interventions^**§**^	
Awareness of assignment	11a	Who was aware of intervention assignment after allocation (for example, participants, providers, those assessing outcomes), and how any masking was done	
	11b	If relevant, description of the similarity of interventions	
Analytical methods	12a	Statistical methods used to compare group outcomes^**§**^	How missing data were handled, with details of any imputation method
	12b	Methods for additional analyses, such as subgroup analyses, adjusted analyses, and process evaluations	
Results			
Participant flow (a diagram is strongly recommended)	13a	For each group, the numbers randomly assigned, receiving the intended intervention, and analysed for the outcomes^**§**^	Where possible, the number approached, screened, and eligible prior to random assignment, with reasons for non-enrolment
	13b	For each group, losses and exclusions after randomisation, together with reasons^**§**^	
Recruitment	14a	Dates defining the periods of recruitment and follow-up	
	14b	Why the trial ended or was stopped	
Baseline data	15	A table showing baseline characteristics for each group^**§**^	Include socioeconomic variables where applicable
Numbers analysed	16	For each group, number included in each analysis and whether the analysis was by original assigned groups^**§**^	
Outcomes and estimation	17a	For each outcome, results for each group, and the estimated effect size and its precision (such as 95% confidence interval)^**§**^	Indicate availability of trial data
	17b	For binary outcomes, presentation of both absolute and relative effect sizes is recommended	
Ancillary analyses	18	Results of any other analyses performed, including subgroup analyses, adjusted analyses, and process evaluations, distinguishing pre-specified from exploratory	
Harms	19	All important harms or unintended effects in each group (for specific guidance, see CONSORT for Harms)	
Discussion			
Limitations	20	Trial limitations, addressing sources of potential bias, imprecision, and, if relevant, multiplicity of analyses	
Generalisability	21	Generalisability (external validity, applicability) of the trial findings^**§**^	
Interpretation	22	Interpretation consistent with results, balancing benefits and harms, and considering other relevant evidence	
Important information			
Registration	23	Registration number and name of trial registry	
Protocol	24	Where the full trial protocol can be accessed, if available	
Declaration of interests	25	Sources of funding and other support, role of funders	Declaration of any other potential interests
Stakeholder involvement	26a		Any involvement of the intervention developer in the design, conduct, analysis, or reporting of the trial
	26b		Other stakeholder involvement in trial design, conduct, or analyses
	26c		Incentives offered as part of the trial

This table lists items from the CONSORT 2010 checklist (with some modifications for social and psychological intervention trials as described in Table 2) and additional items in the CONSORT-SPI 2018 extension. Empty rows in the ‘CONSORT-SPI 2018’ column indicate that there is no extension to the CONSORT 2010 item

We strongly recommended that the CONSORT-SPI 2018 Explanation and Elaboration (E&E) document be reviewed when using the CONSORT-SPI 2018 checklist for important clarifications of each item

§An extension item for cluster trials exists for this CONSORT 2010 item

**Table 2:** Noteworthy changes to CONSORT 2010 items in the CONSORT-SPI 2018 checklist

• Item 6a. The distinction between ‘primary’ versus ‘secondary’ outcomes has been removed. • Item 11. ‘Blinding’ has been changed to ‘Awareness of assignment’ and ‘masking’ in the section heading and item wording, respectively. These changes address concerns about the use of the term ‘blinding’ as well as the need to emphasise the issue of awareness of assignment by providers and participants in social and psychological intervention trials. • Item 12. The section heading ‘Statistical methods’ has been changed to ‘Analytical methods’ because some methods may be qualitative in social and psychological intervention RCTs. • Item 12a. The distinction between ‘primary’ versus ‘secondary’ outcomes has been removed. • Item 12b. Process evaluations are specifically highlighted. • Item 13a. The distinction between ‘primary’ versus ‘secondary’ outcomes has been removed. • Items 13a and 16. The wording ‘number of participants’ has been changed to ‘number’ because the term ‘participants’ is not appropriate for RCTs in which the unit of intervention is a geographic area. While social and psychological interventions may target individual participants or groups of individuals such as families or schools, they may also involve place-based techniques that target geographic units and examine area-level effects. However, for convenience and consistency with the CONSORT 2010 guidance [43], the CONSORT-SPI 2018 checklist and E&E will refer to the unit targeted by the intervention as ‘participants’, though ‘participants’ throughout this guidance is meant to stand for ‘participating units’, or the unit being targeted by the intervention [44], which may include geographic units. • Item 15. The words ‘clinical and demographic’ have been removed because this checklist targets interventions that may not be medical in nature or have health outcomes, and thus to emphasise the need to report important baseline characteristics irrespective of their nature. • Item 16. The parenthetical ‘(denominator)’ has been removed. The term implied the use of dichotomous outcomes, whereas continuous outcomes are extremely prevalent in social and psychological intervention RCTs. • Item 17a. The distinction between ‘primary’ versus ‘secondary’ outcomes has been removed. • Items 23–25. The section ‘Other Information’ has been changed to ‘Important Information’ because consensus meeting participants had concerns that ‘Other’ makes the requested information appear to be of secondary importance to previous sections. • Item 25. The phrase ‘such as supply of drugs’ has been removed because drug trials are not in the purview of this extension by definition. • Item 26: New item. A new sub-section in ‘Important Information’ called ‘Stakeholder Involvement’ has been added because consensus meeting participants thought such a sub-section would best fit the three sub-items currently allocated to it	

## Results and Discussion

### Explanation and elaboration of the CONSORT-SPI

#### Title and abstract

##### Item 1a: identification as a randomised trial in the title

Placing the word ‘randomised’ in the title increases the likelihood that an article will be indexed correctly in bibliographic databases and retrieved in electronic searches [45]. Authors should consider providing information in the title to assist interested readers, such as the name of the intervention and the problem that the trial addresses [46]. We advise authors to avoid uninformative titles (e.g. catchy phrases or allusions) because they reduce space that could be used to help readers identify relevant manuscripts [45, 46].

##### Item 1b: structured summary of trial design, methods, results, and conclusions (for specific guidance, see CONSORT for Abstracts) [13, 47]

Abstracts are the most widely read section of manuscripts [46], and they are used for indexing reports in electronic databases [45]. Authors should follow the CONSORT Extension for Abstracts, which provides detailed advice for structured journal article abstracts and for conference abstracts. We have tailored the CONSORT Extension for Abstracts for social and psychological intervention trials with relevant items for the objective, trial design, participants, and interventions from the CONSORT-SPI 2018 checklist (Table 3 and Additional file 3) [47].

**Table 3:** Items to report in journal or conference abstracts for social and psychological intervention trials [13]

Section	CONSORT Abstract item	Relevant CONSORT-SPI item
Title	Identification of the study as randomised	
Authors	Contact details for the corresponding author	
Trial design	Description of the trial design (e.g. parallel, cluster, noninferiority)	If the unit of random assignment is not the individual, refer to CONSORT for Cluster Randomised Trials and report the items included in its extension for abstracts [8]
Methods		
Participants	Eligibility criteria for participants and the settings where the data were collected	When applicable, eligibility criteria for the setting of intervention delivery and the eligibility criteria for the persons who delivered the interventions
Interventions	Interventions intended for each group	
Objective	Specific objective or hypothesis	If pre-specified, how the intervention was hypothesised to work
Outcomes	Clearly defined primary outcome for this report	
Randomisation	How participants were allocated to interventions	
Awareness of assignment	Who was aware of intervention assignment after allocation (for example, participants, providers, those assessing outcomes), and how any masking was done	
Results		
Number randomly assigned	Number randomised to each group	
Recruitment	Trial status	
Interventions		Extent to which interventions were actually delivered by providers and taken up by participants as planned
Number analysed	Number analysed in each group	
Outcomes	For the primary outcome, a result for each group and the estimated effect size and its precision	
Harms	Important adverse events or side effects	
Conclusions	General interpretation of the results	
Trial registration	Registration number and name of trial register	
Funding	Source of funding	

#### Introduction

##### Item 2a: scientific background and explanation of rationale

A structured introduction should describe the rationale for the trial and how the trial contributes to what is known [48]. In particular, the introduction should describe the targeted problem or issue [49] and what is already know about the intervention, ideally by referencing systematic reviews [46].

##### Item 2b: specific objectives or hypotheses

The objectives summarise the research questions, including any hypotheses about the expected magnitude and direction of intervention effects [48, 50]. For social and psychological interventions that have multiple units of intervention and multiple outcome assessments (e.g. individuals, groups, places), authors should specify to whom or to what each objective and hypothesis applies.

#### CONSORT-SPI 2018 item 2b: if pre-specified, how the intervention was hypothesised to work

Describing how the interventions in all groups (i.e. all experimental and comparator groups) were expected to affect outcomes provides important information about the theory underlying the interventions [46]. For each intervention evaluated, authors should describe the 'mechanism of action' [51], also known as the 'theory of change' [52], 'programme theory' [53], or 'causal pathway' [54]. Authors should state how interventions were thought to affect outcomes *prior* to the trial, and whether the hypothesised mechanisms of action were specified *a priori*, ideally with reference to the trial registration and protocol [55]. Specifically, authors should report: how the components of each intervention were expected to influence modifiable psychological and social processes, how influencing these processes was thought to affect the outcomes of interest, the role of context, facilitators of and barriers to intervention implementation, and potential adverse events or unintended consequences [48, 56]. Graphical depictions—such as a logic model or analytic framework—may be useful [19].

#### Methods: trial design

##### Item 3a: description of trial design (such as parallel, factorial) including allocation ratio

Unambiguous details about trial design help readers assess the suitability of trial methods for addressing trial objectives [46, 57], and clear and transparent reporting of all design features of a trial facilitates reproducibility and replication [58]. Authors should explain their choice of design (especially if it is not an individually randomised, two-group parallel trial) [9]; state the allocation ratio and its rationale; and indicate whether the trial was designed to assess the superiority, equivalence, or noninferiority of the interventions [10, 59, 60].

#### CONSORT-SPI 2018 item 3a: if the unit of random assignment is not the individual, please refer to CONSORT for Cluster Randomised Trials

Randomising at the cluster level (e.g. schools) has important implications for trial design, analysis, inference, and reporting. For cluster randomised trials, authors should follow the CONSORT Extension to Cluster Randomised Trials. Because many social and psychological interventions are cluster randomised, we provide the extended items from this checklist in Tables 4 and 5 [8]. Authors should also report the unit of randomisation, which might be social units (e.g. families), organisations (e.g. schools, prisons), or places (e.g. neighbourhoods), and specify the unit of each analysis, especially when the unit of analysis is not the unit of randomisation (e.g. randomising at the cluster level and analysing outcomes assessed at the individual level).

**Table 4:** Items to report in the abstract for cluster randomised social and psychological intervention trials [8]

Section	CONSORT Abstract item	Relevant CONSORT Cluster extension item
Title	Identification of the study as randomised	Identification of study as cluster randomised
Authors	Contact details for the corresponding author	
Trial design	Description of the trial design (e.g. parallel, cluster, noninferiority)	
Methods		
Participants	Eligibility criteria for participants and the settings where the data were collected	Eligibility criteria for clusters
Interventions	Interventions intended for each group	
Objective	Specific objective or hypothesis	Whether objective or hypothesis pertains to the cluster level, the individual participant level, or both
Outcomes	Clearly defined primary outcome for this report	Whether the primary outcome pertains to the cluster level, the individual participant level, or both
Randomisation	How participants were allocated to interventions	How clusters were allocated to interventions
Awareness of assignment	Who was aware of intervention assignment after allocation (for example, participants, providers, those assessing outcomes), and how any masking was done	
Results		
Number randomly assigned	Number of participants randomised to each group	Number of clusters randomised to each group
Recruitment	Trial status	
Number analysed	Number of participants analysed in each group	Number of clusters analysed in each group
Outcomes	For the primary outcome, a result for each group and the estimated effect size and its precision	Results at the cluster or individual level as applicable for each primary outcome
Harms	Important adverse events or side effects	
Conclusions	General interpretation of the results	
Trial registration	Registration number and name of trial register	
Funding	Source of funding

**Table 5:** Items to report in the main text for cluster randomised social and psychological intervention trials [8]

Section	Item #	Cluster extension item
Title	1a	Identification as a cluster randomised trial in the title
Abstract	1b	See Table 4
Introduction		
Background and objectives	2a	Rationale for using a cluster design
	2b	Whether objectives pertain to the cluster level, the individual participant level, or both
Methods		
Trial design	3a	Definition of cluster and description of how the design features apply to the clusters
Participants	4a	Eligibility criteria for clusters
Interventions	5	Whether interventions pertain to the cluster level, the individual participant level, or both
Outcomes	6a	Whether outcome measures pertain to the cluster level, the individual participant level, or both
Sample size	7a	Method of calculation, number of clusters(s) (and whether equal or unequal cluster sizes are assumed), cluster size, a coefficient of intracluster correlation (ICC or *k*), and an indication of its uncertainty
Randomisation		
Sequence generation	8b	Details of stratification or matching if used
Allocation concealment mechanism	9	Specification that allocation was based on clusters rather than individuals and whether allocation concealment (if any) was at the cluster level, the individual participant level, or both
Implementation	10a	Who generated the random allocation sequence, who enrolled clusters, and who assigned clusters to interventions
	10b	Mechanism by which individual participants were included in clusters for the trial (such as complete enumeration, random sampling)
	10c	From whom consent was sought (representatives of the cluster, individual cluster members, or both) and whether consent was sought before or after randomisation
Analytical methods	12a	How clustering was taken into account
Results		
Participant flow (a diagram is strongly recommended)	13a	For each group, the numbers of clusters that were randomly assigned, received the intended treatment, and were analysed for the primary outcome
	13b	For each group, losses and exclusions for both clusters and individual cluster members
Baseline data	15	Baseline characteristics for the individual and cluster levels as applicable for each group
Numbers analysed	16	For each group, number of clusters included in each analysis
Outcomes and estimation	17a	Results at the individual or cluster level as applicable and a coefficient of intracluster correlation (ICC or *k*) for each primary outcome
Generalisability	21	Generalisability to clusters and/or individual participants (as relevant)

##### Item 3b: important changes to methods after trial commencement (such as eligibility criteria), with reasons

Deviations from planned trial methods are common, and not necessarily associated with flawed or biased research. Changes from the planned methods are important for understanding and interpreting trial results. A trial report should refer to a trial registration (Item 23) [61–63] and protocol (Item 24) developed in advance of assigning the first participant [55], and to a pre-specified statistical analysis plan [64]. The report should summarise all amendments to the protocol and statistical analysis plan, when they were made, and the rationale for each amendment. Because selective outcome reporting is pervasive [65], authors should state any changes to the outcome definitions during the trial.

#### Methods: participants

##### Item 4a: eligibility criteria for participants

Eligibility criteria should describe how participants (i.e. individuals, groups, or places) were recruited. Readers need this information to understand who could have entered the trial and the generalisability of findings. Authors should describe all inclusion and exclusion criteria used to determine eligibility, as well as the methods used to screen and assess participants to determine their eligibility [46, 48].

#### CONSORT-SPI 2018 item 4a: when applicable, eligibility criteria for settings and those delivering the interventions

In addition to the eligibility criteria that apply to individuals, social and psychological intervention trials often have eligibility criteria for the settings where participants will be recruited and interventions delivered, as well as intervention providers [44]. Authors should describe these criteria to help readers compare the trial context with other contexts in which interventions might be used [48, 66, 67].

##### Item 4b: settings and locations of intervention delivery and where the data were collected

Information about settings and locations of intervention delivery and data collection are essential for understanding trial context. Important details might include the geographic location, day and time of trial activities, space required, and features of the inner setting (e.g. implementing organisation) and outer setting (e.g. external context and environment) that might influence implementation [68]. Authors should refer to the mechanism of action when deciding what information about setting and location to report.

#### Methods: interventions

##### Item 5: the interventions for each group with sufficient details to allow replication, including how and when they were actually administered

Complete and transparent information about the content and delivery of all interventions in all groups (experimental and comparator) [44] is vital for understanding, replicating, and synthesising intervention effects [54, 69]. Essential information includes: naming the interventions, what was actually delivered (e.g. materials and procedures), who provided the interventions, how, where, when, and how much [70]. Details about providers should include their professional qualifications and education, expertise or competence with the interventions or area in general, and training and supervision for delivering the interventions [71]. Tables or diagrams showing the sequence of intervention activities, such as the participant timeline recommended in the Standard Protocol Items: Recommendations for Interventional Trials (SPIRIT) 2013 Statement [55], are often useful [72]. Authors should avoid the sole use of labels such as ‘treatment as usual’ or ‘standard care’ because they are not uniform across time and place [48].

#### CONSORT-SPI 2018 item 5a: extent to which interventions were actually delivered by providers and taken up by participants as planned

Frequently, interventions are not implemented as planned. Authors should describe the *actual delivery* by providers and *uptake* by participants of interventions for all groups, including methods used to ensure or assess whether the interventions were delivered by providers and taken up by participants as intended [69]. Quantitative or qualitative process evaluations [51] may be used to assess what providers actually did (e.g. recording and coding sessions), the amount of an intervention that participants received (e.g. recording the number of sessions attended), and contamination across intervention groups [73, 74]. Authors should distinguish planned systematic adaptations (e.g. tailoring) from modifications that were not anticipated in the trial protocol. When this information cannot be included in a single manuscript, authors should use online supplements, additional reports, and data repositories to provide this information.

#### CONSORT-SPI 2018 item 5b: where other informational materials about delivering the interventions can be accessed

Authors should indicate where readers can find sufficient information to replicate the interventions, such as intervention protocols [75], training manuals [48], or other materials (e.g. worksheets and websites) [54]. For example, new online platforms such as the Open Science Framework allow researchers to share some or all of their study materials freely (https://osf.io).

#### CONSORT-SPI 2018 item 5c: when applicable, how intervention providers were assigned to each group

Some trials assign specific providers to different conditions to prevent expertise and allegiance from confounding the results. Authors should report whether the same people delivered the experimental and comparator interventions, whether providers were nested within intervention groups, and the number of participants assigned to each provider.

#### Methods: outcomes

##### Item 6a: completely define pre-specified outcomes, including how and when they were assessed

All outcomes should be defined in sufficient detail for others to reproduce the results using the trial data [50, 76]. An outcome definition includes: (1) the domain (e.g. depression), (2) the measure (e.g. the Beck Depression Inventory II Cognitive subscale), (3) the specific metric (e.g. a value at a time point, a change from baseline), (4) the method of aggregation (e.g. mean, proportion), and (5) the time point (e.g. 3 months post-intervention) [50]. In addition, authors should report the methods and persons used to collect outcome data, properties of measures or references to previous reports with this information, methods used to enhance measurement quality (e.g. training of outcome assessors), and any differences in outcome assessment between trial groups [46]. Authors also should indicate where readers can access materials used to measure outcomes [24]. When a trial includes a measure (e.g. a questionnaire) that is not available publicly, authors should provide a copy (e.g. through an online repository or as an online supplement).

##### Item 6b: any changes to trial outcomes after the trial commenced, with reasons

All outcomes assessed should be reported. If the reported outcomes differ from those in the trial registration (Item 23) or protocol (Item 24), authors should state which outcomes were added and which were removed. To allow readers to assess the risk of bias from outcome switching, authors should also identify any changes to level of importance (e.g. primary or secondary) [77]. Authors should provide the rationale for any changes made and state whether these were done before or after collecting the data.

#### Methods: sample size

##### Item 7a: how sample size was determined

Authors should indicate the intended sample size for the trial and how it was determined, including whether the sample size was determined *a priori* using a sample size calculation or due to practical constraints. If an *a priori *sample size calculation was conducted, authors should report the effect estimate used for the sample size calculation and why it was chosen (e.g. the smallest effect size of interest, from a meta-analysis of previous trials). If an *a priori* sample size calculation was not performed, authors should not present a post hoc calculation, but rather the genuine reason for the sample size (e.g. limitations in time or funding) and the actual power to detect an effect for each result (Item 17).

##### Item 7b: when applicable, an explanation of any interim analyses and stopping guidelines

Multiple statistical analyses can lead to false-positive results, especially when using stopping guidelines based on statistical significance. Any interim analyses should be described, including which analyses were conducted (i.e. the outcomes and methods of analysis), when they were conducted, and why (particularly whether they were pre-specified [78]). Authors should also describe the reasons for stopping the trial, including any procedures used to determine whether the trial would be stopped early (e.g. regular meetings of a data safety monitoring board) [79].

#### Methods: randomisation—sequence generation

##### Item 8a: method used to generate the random allocation sequence

In a randomised trial, participants are assigned to groups by chance using processes designed to be unpredictable. Authors should describe the method used to generate the allocation sequence (e.g. a computer-generated random number sequence), so that readers may assess whether the process was truly random. Authors should not use the term ‘random’ to describe sequences that are deterministic (e.g. alternation, order of recruitment, date of birth).

##### Item 8b: type of randomisation; details of any restriction (such as blocking and block size)

Some trials restrict randomisation to balance groups in size or important characteristics. Blocking restricts randomisation by grouping participants into 'blocks' and by assigning participants using a random sequence within each block [46]. When blocking is used, authors should describe how the blocks were generated, the size of the blocks, whether and how block size varied, and if trial staff became aware of the block size. Stratification restricts randomisation by creating multiple random allocation sequences based on site or characteristics thought to modify intervention effects [46]. When stratification is used, authors should report why it was used and describe the variables used for stratification, including cut-off values for categories within each stratum. When minimisation is used, authors should report the variables used for minimisation and include the statistical code. When there are no restrictions on randomisation, authors should state that they used ‘simple randomisation’ [43].

#### Methods: randomisation—allocation concealment mechanism

##### Item 9: mechanism used to implement the random allocation sequence, describing any steps taken to conceal the sequence until interventions were assigned

In addition to *generating* a truly random sequence (Item 8a), researchers should *conceal* the sequence to prevent foreknowledge of the intervention assignment by persons enrolling and assigning participants. Otherwise, recruitment and allocation could be affected by knowledge of the next assignment. Authors should report whether and how allocation was concealed [80, 81]. When allocation was concealed, authors should describe the mechanism and how this mechanism was monitored to avoid tampering or subversion (e.g. centralised or 'third-party' assignment, automated assignment system, sequentially numbered identical containers, sealed opaque envelopes). While masking (blinding) is not always possible, allocation concealment is always possible.

#### Methods: randomisation—implementation

##### Item 10: who generated the random allocation sequence, who enrolled participants, and who assigned participants to interventions

In many individually randomised trials, staff who generate and conceal the random sequence are different from the staff involved in implementing the sequence. This can prevent tampering or subversion [48]. Other procedures may be used to ensure true randomisation in trials in which participants (e.g. groups, places) are recruited and then randomised at the same time. Authors should indicate who carried out each procedure (i.e. generating the random sequence, enrolling participants, and assigning participants to interventions) and the methods used to protect the sequence.

#### Methods: awareness of assignment

##### Item 11a: who was aware after assignment to interventions (for example, participants, providers, those assessing outcomes), and how any masking was done

Masking (blinding) refers to withholding information about assigned interventions *post-randomisation* from those involved in the trial [46]. Masking can reduce threats to internal validity arising from an awareness of the intervention assignment by those who could be influenced by this knowledge. Authors should state whether and how (a) participants, (b) providers, (c) data collectors, and (d) data analysts were kept unaware of intervention assignment. If masking was not done (e.g. because it was not possible), authors should describe the methods, if any, used to assess performance and expectancy biases (e.g. masking trial hypotheses, measuring participant expectations) [82]. Although masking of providers and participants is often not possible, masking outcome assessors is usually possible, even for outcomes assessed through interviews or observations. If examined, authors should report the extent to which outcome assessors remained masked to participants’ intervention status.

##### Item 11b: if relevant, description of the similarity of interventions

Particularly because masking providers and participants is impossible in many social and psychological intervention trials, authors should describe any differences between interventions delivered to each group that could lead to differences in the performance and expectations of providers and participants. Important details include differences in intervention components and acceptability, co-interventions (or adjunctive interventions) that might be available to some groups and not others, and contextual differences between groups (e.g. differences in place of delivery).

#### Methods: analytical methods

##### Item 12a: statistical methods used to compare group outcomes

Complete statistical reporting allows the reader to understand the results and to reproduce analyses [48]. For each outcome, authors should describe the methods of analysis, including transformations and adjustment for covariates, and whether the methods of analysis were chosen *a priori* or decided after data were collected. In the United States, trials funded by the National Institutes of Health must deposit a statistical analysis plan on www.ClinicalTrials.gov with their results [62, 63]. Authors with other funding sources should ascertain whether there are similar requirements. For cluster randomised trials, authors should state whether the unit analysed differs from the unit of assignment, and if applicable, the analytical methods used to account for differences between the unit of assignment, level of intervention, and the unit of analysis [8, 44, 46]. Authors should also note any procedures and rationale for any transformations to the data [46]. To facilitate full reproducibility, authors should report software used to run analyses and provide the exact statistical code [24].

#### Extended CONSORT-SPI item 12a: how missing data were handled, with details of any imputation method

Missing data are common in trials of social and psychological interventions for many reasons, such as participant discontinuation, missed visits, and participant failure to complete all items or measures (even for participants who have not discontinued the trial) [46]. Authors should report the amount of missing data, evidence regarding the reasons for missingness, and assumptions underlying judgements about missingness (e.g. missing at random) [83]. For each outcome, authors should describe the analysis population (i.e. participants who were eligible to be included in the analysis) and the methods for handling missing data, including procedures to account for missing participants (i.e. participants who withdrew from the trial, did not complete an assessment, or otherwise did not provide data) and procedures to account for missing data items (i.e. questions that were not completed on a questionnaire) [76]. Imputation methods, which aim to estimate missing data based on other data in the dataset, can influence trial results [84]. When imputation is used, authors should describe the variables used for imputation, the number of imputations performed, the software procedures for executing the imputations, and the results of any sensitivity analyses conducted to test assumptions about missing data [84]. For example, it is often helpful to report results without imputation to help readers evaluate the consequences of imputing data.

##### Item 12b: methods for additional analyses, such as subgroup analyses, adjusted analyses, and process evaluations

In addition to analysing impacts on primary and secondary outcomes, trials often include additional analyses, such as subgroup analyses and mediation analyses to investigate processes of change [51, 85]. All analyses should be reported at the same level of detail. Authors should indicate which subgroup analyses were specified *a priori* in the trial registration or protocol (Items 23 and 24), how subgroups were constructed, and distinguish confirmatory analyses from exploratory analyses. For adjusted analyses, authors should report the statistical procedures and covariates used and the rationale for these.

Additionally, qualitative analyses may be used to investigate processes of change, implementation processes, contextual influences, and unanticipated outcomes [51]. Authors should indicate whether such analyses were undertaken or are planned (and where they are or will be reported if so). Authors should report methods and results of qualitative analyses according to reporting standards for primary qualitative research [86].

#### Results: participant flow

**Item 13a: for each group, the numbers randomly assigned, receiving intended treatment, and analysed for the outcomes**

#### CONSORT-SPI item 13a: where possible, the number approached, screened, and eligible prior to random assignment, with reasons for non-enrolment

Attrition after randomisation can affect internal validity (i.e. by introducing selection bias), and attrition before or after randomisation can affect generalisability [46]. Authors should report available information about the total number of participants at each stage of the trial, with reasons for non-enrolment (i.e. before randomisation) or discontinuation (i.e. after randomisation). Key stages typically include: approaching participants, screening for potential eligibility, assessment to confirm eligibility, random assignment, intervention receipt, and outcome assessment. As there may be delays between each stage (e.g. between randomisation and initiation of the intervention) [87], authors should include a flow diagram to describe trial attrition in relation to each of these key stages (Fig. 1; Additional file 4)

##### Item 13b: for each group, losses and exclusions after randomisation, together with reasons

Authors should report participant attrition and data exclusion by the research team for each randomised group at each follow-up point [48]. Authors should distinguish between the number of participants who deviate from the intervention protocol but continue to receive an intervention, discontinue an intervention but continue to provide outcome data, discontinue the trial altogether, and were excluded by the investigators. Authors should provide reasons for each loss (e.g. lost contact, died) and exclusion (e.g. excluded by the investigators because of poor adherence to intervention protocol), and indicate the number of persons who discontinued for unknown reasons.

#### Results: recruitment

##### Item 14a: dates defining the periods of recruitment and follow-up

The dates of a trial and its activities provide readers some information about the historical context of the trial [48]. The SPIRIT 2013 Statement includes a table that authors can use to provide a complete schedule of trial activities, including recruitment practices, pre-randomisation assessments, periods of intervention delivery, a schedule of post-randomisation assessments, and when the trial was stopped [55]. In the description, authors should define baseline assessment and follow-up times relative to randomisation. For example, by itself, ‘4-week follow-up’ is unclear and could mean different things if meant after randomisation or after the end of an intervention.

##### Item 14b: why the trial ended or was stopped

Authors should state why the trial was stopped. Trials might be stopped for reasons decided *a priori* (e.g. sample size reached and predetermined follow-up period completed) or in response to the results. For trials stopped early in response to interim analyses (Item 7b), authors should state the reason for stopping (e.g. for safety or futility) and whether the stopping rule was decided *a priori*. If applicable, authors should describe other reasons for stopping, such as implementation challenges (e.g. could not recruit enough participants) or extrinsic factors (e.g. a natural disaster). Authors should indicate whether there are plans to continue collecting outcome data (e.g. long-term follow-up).

#### Results: baseline data

**Item 15: a table showing baseline characteristics for each group**

#### CONSORT-SPI item 15: include socioeconomic variables where applicable

Authors should provide a table summarising all data collected at baseline, with descriptive statistics for each randomised group. This table should include all important characteristics measured at baseline, including pre-intervention data on trial outcomes, and potential prognostic variables. Authors should pay particular attention to topic-specific information related to socioeconomic and other inequalities [88–90]. For continuous variables, authors should report the average value and its variance (e.g. mean and standard deviation). For categorical variables, authors should report the numerator and denominator for each category. Authors should not use standard errors and confidence intervals for baseline data because these are inferential (rather than descriptive): inferential statistics assess the probability that observed differences occurred by chance, and all baseline differences in randomised trials occur by chance [91].

#### Results: numbers analysed

##### Item 16: for each group, number included in each analysis and whether the analysis was by original assigned groups

While a flow diagram is helpful for indicating the number of participants at each trial stage, the number of participants included in each analysis often differs across outcomes and analyses [44]. Authors should report the number of participants per intervention group for each analysis, so readers can interpret the results and perform secondary analyses of the data. For each outcome, authors should also identify the analysis population and the method used for handling missing data (Item 12a) [76].

#### Results: outcomes and estimation

##### Item 17a: for each outcome, results for each group, and the estimated effect size and its precision (such as 95% confidence interval)

For each outcome in a trial, authors should report summary results for all analyses, including results for each trial group and the contrast between groups, the estimated magnitude of the difference (effect size), the precision or uncertainty of the estimate (e.g. 95% confidence interval or CI), and the number of people included in the analysis in each group. The *p* value does *not* describe the precision of an effect estimate, and authors should report precision even if the difference between groups is not statistically significant [46]. For categorical outcomes, summary results for each analysis should include the number of participants with the event of interest. The effect size can be expressed as the risk ratio, odds ratio, or risk difference and its precision (e.g. 95% CI). For continuous outcomes, summary results for each analysis should include the average value and its variance (e.g. mean and standard error). The effect size is usually expressed as the mean difference and its precision (e.g. 95% CI). Summary results are often more clearly presented in a table rather than narratively in text.

#### CONSORT-SPI item 17a: indicate availability of trial data

As part of the growing open-science movement, triallists are increasingly expected to maintain their datasets, linked via trial registrations and posted in trusted online repositories (see http://www.bitss.org/resource-tag/data-repository/), to facilitate reproducibility of reported analyses and future secondary data analyses. Data sharing is also associated with higher citations [92]. Authors should indicate whether and how to obtain trial datasets, including any metadata and analytic code needed to replicate the reported analyses [24, 93]. Any legal or ethical restrictions on making the trial data available should be described [93].

##### Item 17b: for binary outcomes, presentation of both absolute and relative effect sizes is recommended

By themselves, neither relative measures nor absolute measures provide comprehensive information about intervention effects. Authors should report relative effect sizes (e.g. risk ratios) to express the strength of effects and absolute effect sizes (e.g. risk differences) to indicate actual differences in events between interventions [94].

#### Results: ancillary analyses

##### Item 18: results of any other analyses performed, including subgroup analyses, adjusted analyses, and process evaluations, distinguishing pre-specified from exploratory

Authors should report the results for each additional analysis described in the methods (Item 12b), indicating the number of analyses performed for each outcome, which analyses were pre-specified, and which analyses were not pre-specified. When evaluating effects for subgroups, authors should report interaction effects or other appropriate tests for heterogeneity between groups, including the estimated difference in the intervention effect between each subgroup with confidence intervals. Comparing tests for the significance of change *within* subgroups is not an appropriate basis for evaluating differences *between* subgroups. If reporting adjusted analyses, authors should provide unadjusted results as well. Authors reporting any results from qualitative data analyses should follow reporting standards for qualitative research [86], though adequately reporting these findings will likely require more than one journal article [51].

#### Results: harms

##### Item 19: all important harms or unintended effects in each group (for specific guidance, see CONSORT for Harms) [14]

Social and psychological interventions have the potential to produce unintended effects, both harmful and beneficial [95]. These may be identified in the protocol and relate to the theory of how the interventions are hypothesised to work (Item 2b) [56], or they may be unexpected events that were not pre-specified for assessment. Harms may include indirect effects such as increased inequalities at the level of groups or places that result from the intervention [89]. When reporting quantitative data on unintended effects, authors should indicate how they were defined and measured, and the frequency of each event per trial group. Authors should report all results from qualitative investigations that identify possible unintended effects because this information may help readers make informed decisions about using interventions in future research and practice.

#### Discussion: limitations

##### Item 20: trial limitations, addressing sources of potential bias, imprecision, and, if relevant, multiplicity of analyses

Authors should provide a balanced discussion of the strengths and limitations of the trial and its results. Authors should consider issues related to risks of bias, precision of effect estimates, the use of multiple outcomes and analyses, and whether the intervention was delivered and taken up as planned.

#### Discussion: generalisability

##### Item 21: generalisability (external validity, applicability) of the trial findings

Authors should address generalisability, or the extent to which the authors believe that trial results can be expected in other situations [66]. Authors should explain how statements about generalisability relate to the trial design and execution. Key factors to consider discussing include: recruitment practices, eligibility criteria, sample characteristics, facilitators and barriers to intervention implementation, the choice of comparator, what outcomes were assessed and how, length of follow-up, and setting characteristics [46, 66].

#### Discussion: interpretation

##### Item 22: interpretation consistent with results, balancing benefits and harms, and considering other relevant evidence

Authors should provide a brief interpretation of findings in light of the trial’s objectives or hypotheses [46]. Authors may wish to discuss plausible alternative explanations for results other than differences in effects between interventions [44]. Authors should contextualise results and identify the additional knowledge gained by discussing how the trial adds to the results of other relevant literature [96], including references to previous trials and systematic reviews. If theory was used to inform intervention development or evaluation (Item 2b), authors should discuss how the results of the trial compare with previous theories about how the interventions would work [97, 98]. Authors should consider describing the practical significance of findings; the potential implications of findings to theory, practice and policy; and specific areas of future research to address gaps in current knowledge [49]. Authors should avoid distorted presentation or 'spin' when discussing trial findings [99, 100].

#### Important information: registration

##### Item 23: registration number and name of trial registry

Trial registration is the posting of a minimum information set in a public database, including: eligibility criteria, all outcomes, intervention protocols, and planned analyses [101]. Trial registration aids systematic reviews and meta-analyses, and responds to decades-long calls to prevent reporting biases [102–104]. Trial registration is now required for all trials published by journals that endorse the International Committee of Medical Journal Editors guidelines and for all trials funded by the National Institutes of Health in the United States as well as the Medical Research Council and National Institute for Health Research in the UK [62, 63, 105, 106].

Trials should be registered *prospectively*, before beginning enrolment, normally in a publicly accessible website managed by a registry conforming to established standards [101, 105]. Authors should report the name of the trial registry, the unique identification number for the trial provided by that registry, and the stage at which the trial was registered. If authors did not register their trial, they should report this and the reason for not registering. Registries used in clinical medicine (e.g. www.ClinicalTrials.gov) are suitable for social and psychological intervention trials with health outcomes [107, 108], and several registries exist specifically for social and psychological interventions (http://www.bitss.org/resource-tag/registry/).

#### Important information: protocol

##### Item 24: where the full trial protocol can be accessed, if available

Details about trial design should be described in a publicly accessible protocol (e.g. published manuscript, report in a repository) that includes a record of all amendments made after the trial began. Authors should report where the trial protocol can be accessed. Guidance on developing and reporting protocols has recently been published [55]. Authors of social and psychological intervention trials who face difficulty finding a journal that publishes trial protocols could search for journals supporting the Registered Reports format (https://cos.io/rr/) or upload their trial protocols to relevant preprint servers such as PsyArXiv (https://psyarxiv.com/) and SocArXiv (https://osf.io/preprints/socarxiv).

#### Important information: funding

##### Item 25a: sources of funding and other support, role of funders

Information about trial funding and support is important in helping readers to identify potential conflicts of interest. Authors should identify and describe all sources of monetary or material support for the trial, including salary support for trial investigators and resources provided or donated for any phase of the trial (e.g. space, intervention materials, assessment tools). Authors should report the name of the persons or entities supported, the name of the funder, and the award number. They should also specifically state if these sources had any role in the design, conduct, analysis, and reporting of the trial, and the nature of any involvement or influence. If funders had no involvement or influence, authors should specifically report this.

#### CONSORT-SPI item 25b: declaration of any other potential interests

In addition to financial interests, it is important that authors declare any other potential interests that may be perceived to influence the design, conduct, analysis, or reporting of the trial following established criteria [109]. Examples include allegiance to or professional training in evaluated interventions. Authors should err on the side of caution in declaring potential interests. If authors do not have any financial, professional, personal, or other potential interests to declare, they should declare this explicitly.

### Important information: stakeholder involvement

#### CONSORT-SPI new item – Item 26a: any involvement of the intervention developer in the design, conduct, analysis, or reporting of the trial

Intervention developers are often authors of trial reports. Because involvement of intervention developers in trials may be associated with effect sizes [110, 111], authors should report whether intervention developers were involved in designing the trial, delivering the intervention, assessing the outcomes, or interpreting the data. Authors should also disclose close collaborations with the intervention developers (e.g. being a former student of the developer, serving on an advisory or consultancy board related to the intervention), and any legal or intellectual rights related to the interventions, especially if these could lead to future financial interests.

#### CONSORT-SPI new item – Item 26b: other stakeholder involvement in trial design, conduct, or analyses

Researchers are increasingly called to consult or collaborate with those who have a direct interest in the results of trials, such as providers, clients, and payers [112]. Stakeholders may be involved in designing trials (e.g. choosing outcomes) [76], delivering interventions, or interpreting trial results. Stakeholder involvement may help to better ensure the acceptability, implementability, and sustainability of interventions as they move from research to real-world settings [113]. When applicable, authors should describe which stakeholders were involved, how they were recruited, and how they were involved in various stages of the trial [114]. Authors may find reporting standards on public involvement in research useful [115].

#### CONSORT-SPI new item – Item 26c: incentives offered as part of the trial

Incentives offered to participants, providers, organisations, and others involved in a trial can influence recruitment, engagement with the interventions, and quality of intervention delivery [116]. Incentives include monetary compensation, gifts (e.g. meals, transportation, access to services), academic credit, and coercion (e.g. prison diversion) [48]. When incentives are used, authors should make clear at what trial stage and for what purpose incentives are offered, and what these incentives entail. Authors also should state whether incentives differ by trial group, such as compensation for participants receiving the experimental rather than the comparator interventions.

## Conclusions

The results of RCTs are of optimal use when authors report their methods and results accurately, completely, and transparently. The CONSORT-SPI 2018 checklist can help researchers to design and report future trials, and provide guidance to peer reviewers and editors for evaluating manuscripts, to funders in setting reporting criteria for grant applications, and to educators in teaching trial methods. Each item should be addressed before or within the main trial paper (e.g. in the text, as an online supplement, or by reference to a previous report). The level of detail required for some checklist items will depend on the nature of the intervention being evaluated [70], the trial phase [117], and whether the trial involves an evaluation of process or implementation [74].

CONSORT-SPI 2018, like all CONSORT guidance, is an evolving document with a continuous and iterative process of assessment and refinement over time. The authors welcome feedback about the checklist and this E&E document, particularly as new evidence in this area and greater experience with this guidance develop. For instance, interested readers can provide feedback on whether some of the new items in CONSORT-SPI 2018 that are applicable to other types of trials (e.g. handling missing data and availability of trial data) should be incorporated into the next update of the main CONSORT Statement. We also encourage journals in the behavioural and social sciences to join the hundreds of medical journals that endorse CONSORT guidelines, and to inform us of such endorsement. The ultimate benefit of this collective effort should be better practices leading to better health and quality of life.

## Additional files

Additional file 1: **Table S1.** The CONSORT-SPI group. (DOCX 68 kb)

Additional file 2: **Table S2.** Examples of information to include when reporting randomised trials of social and psychological interventions. (DOCX 132 kb)

Additional file 3: **Table S3.** Examples of abstracts adherent to the CONSORT-SPI Extension for Abstracts. (DOCX 73 kb)

Additional file 4: **Figure S1.** Example of a participant flow diagram. (DOCX 111 kb)

## Abbreviations

CI: Confidence interval
CONSORT: Consolidated Standards of Reporting Trials
CONSORT-SPI: Consolidated Standards of Reporting Trials for Social and Psychological Interventions
E&E: Explanation and Elaboration
RCT: Randomised controlled trial
SPIRIT: Standard Protocol Items: Recommendations for Interventional Trials
UK: United Kingdom
US: United States
