# Supplementary material for: CONSORT-SPI 2018 Explanation and Elaboration: guidance for reporting social and psychological intervention trials
Source: Trials. 2018 Jul 31;19:406. doi: 10.1186/s13063-018-2735-z (PMC6066913; doi:10.1186/s13063-018-2735-z)
Supplement: Supplementary file 1 — Table S1. The CONSORT-SPI group. (DOCX 68 kb) [file 13063_2018_2735_MOESM1_ESM.docx]

**Additional file 1: Table S1. The CONSORT-SPI Group**

The members of the CONSORT-SPI Group were invited to represent the following stakeholder groups at the time of the consensus meeting:

**Project Executive**

Sean Grant RAND Corporation

Sally Hopewell University of Oxford

Evan Mayo-Wilson Johns Hopkins University

Susan Michie University College London

David Moher Ottawa Health Research Institute

Paul Montgomery University of Birmingham

Geraldine Macdonald University of Bristol

**International Advisory Board**

*Stakeholder Representatives of Behavioural and Social Science Disciplines*

J. Lawrence Aber New York University

David Clark University of Oxford

Manuel Eisner University of Cambridge

Frances Gardner University of Oxford

Steve Hollon Vanderbilt University

Lawrence Sherman University of Cambridge

James Thomas UCL Institute of Education

Elizabeth Waters University of Melbourne (Deceased)

Joanne Yaffe University of Utah

*Stakeholder Representatives of Intervention Research Methodologists*

Andrew Booth University of Sheffield

Peter Craig University of Glasgow

Larry Hedges Northwestern University

*Stakeholder Representatives of Journals*

Doug Altman *Trials*

Mark W. Fraser *Journal of the Society for Social Work and Research*

Spyros Konstantopoulos *Journal of Research on Educational Effectiveness*

Kenneth McLeroy *American Journal of Public Health*

Arthur Nezu *Journal of Consulting and Clinical Psychology*

Edmund Sonuga-Barke *Journal of Child Psychology and Psychiatry*

Gary VandenBos *American Psychologist*

Robert West *Addiction*

*Stakeholder Representatives of Research Funders*

Robert Kaplan Office of Behavioral and Social Sciences Research

Peter Kaufmann National Heart, Lung, & Blood Institute

Brian Mittman Patient-Centered Outcomes Research Institute
