# Supplementary material for: CONSORT-SPI 2018 Explanation and Elaboration: guidance for reporting social and psychological intervention trials
Source: Trials. 2018 Jul 31;19:406. doi: 10.1186/s13063-018-2735-z (PMC6066913; doi:10.1186/s13063-018-2735-z)
Supplement: Supplementary file 2 — Table S2. Examples of information to include when reporting randomised trials of social and psychological interventions. (DOCX 132 kb) [file 13063_2018_2735_MOESM2_ESM.docx]

**Additional file 2: Table S2. Examples of information to include when reporting randomised trials of social and psychological interventions**

| **Checklist item** | **Examples for social and psychological intervention trials** | | **Examples for items specific to cluster-randomised trials** | |
| --- | --- | --- | --- | --- |
| **Title and abstract**  Item 1a.  Standard CONSORT description  Identification as a randomised trial in the title**^§^** | Using problem-solving therapy to reduce depressive symptom severity among older adult methadone clients: A randomized clinical trial.^1^ | | Impact of a social-emotional and character development program on school-level indicators of academic achievement, absenteeism, and disciplinary outcomes: A matched-pair, cluster-randomized, controlled trial.^2^ |  |
| Item 1b.  Standard CONSORT description  Structured summary of trial design, methods, results, and conclusions (for specific guidance see CONSORT for abstracts)**^§^**  CONSORT-SPI extension  Refer to CONSORT extension for social and psychological intervention trial abstracts | See Appendix Table 3. | | See Appendix Table 3. |  |
| **Introduction**  Item 2a.  Standard CONSORT description  Scientific background and explanation of rationale**^§^** | National data suggest that 15% to 25% of women will be the victim of an attempted or completed rape during their lifetime. Research suggests college women are at greater risk for sexual victimization than women in the general population. The mental health consequences of sexual assault are serious. Women who are victims of sexual violence have higher and more severe rates of posttraumatic stress disorder (PTSD) than survivors of accidents and natural disasters. In addition to PTSD, there are many other insidious effects of sexual violence, which include psychological distress, physical distress, interpersonal problems, and increased risk for sexual revictimization.^3^ | | Sampling was based on a cluster randomized approach with schools, rather than individuals or classes, as the randomization units in order to minimize possible contamination or spillover effects between treatment conditions.^4^ |  |
| Item 2b.  Standard CONSORT description  Specific objectives or hypotheses**^§^** | We thus hypothesize that sexual safety among HIV-positive men would be facilitated by self-efficacy and skills for enhancing social support and coping with HIV, modulating negative affect, enhancing HIV disclosure, and enhancing information and motivation specifically around sexuality…. We [also] hypothesized that overall unprotected anal intercourse [UAI] would lessen only moderately, whereas transmission risk—UAI that may transmit HIV to uninfected partners—would show significant intervention effects.^5^ | | The central question we addressed is, relative to the “business-as-usual” control condition, what is the effect of assignment to receive the Open Court Reading professional development and curricular materials on the spring literacy achievement outcomes of elementary school classrooms?^6^ |  |
| CONSORT-SPI extension  If pre-specified, how the intervention was hypothesised to work | Parent–infant interaction that is attuned and in which the parent is able to ‘read’ the child's communicative signals promotes positive social and communicative development in all children. Infants at-risk for autism often show ‘weak’ or distorted communicative signals which parents can struggle to recognise and respond to accurately. The iBASIS intervention was designed specifically to reverse such disrupted patterns of early parent–infant interaction, with the hypothesis that there would be consequent positive effects on other infant developmental markers and emerging prodromal autism symptoms…. iBASIS-Video Interaction for Promoting Positive Parenting (iBASIS-VIPP) uses video-feedback to help parents understand and adapt to their infant's individual communication style to promote optimal social and communicative development.^7^ | |  |  |
| **Methods – Trial Design**  Item 3a.  Standard CONSORT description  Description of trial design (such as parallel, factorial) including allocation ratio**^§^**  CONSORT-SPI extension  If the unit of random assignment is not the individual, please refer to CONSORT for Cluster Randomised Trials | We employed a 2 (intervention: CBT (cognitive-behavioural therapy) vs. GHE (general health education)) x 3 (time: end of counseling at 2 weeks, follow-ups at 3 and 6 months) mixed factorial design. Participants … were randomly assigned to receive either CBT or GHE at a 1:1 ratio (*n* = 77 per intervention group).^8^ | | We conducted a stratified randomized pretest–posttest controlled design study by enrolling passive recreation areas (PRAs) within public parks in 3 annual waves. After completion of the pretest assessment, parks were randomized by an independent biostatistician in an unequal 1:3 allocation ratio to treatment (shaded) versus control (unshaded) stratified by city, wave, and pretest use of the study PRA.^9^ |  |
| Item 3b.  Standard CONSORT description  Important changes to methods after trial commencement (such as eligibility criteria), with reasons | Originally, each teacher had three seventh-grade classes participating in the study. Each class was randomly assigned to receive one of the treatments so that each teacher taught all three treatments. The purpose of this restricted random assignment was to reduce the potential confounding effect of instructional methods. However, after the school year started, Teacher C lost one of her seventh-grade classes (the third treatment) because of a change of assignment to teach eighth-grade science.^10^ | |  |  |
| **Methods – Participants**  Item 4a.  Standard CONSORT description  Eligibility criteria for participants**^§^** | Families were referred to the program by schools (30%), community-based agencies (22%), health care clinics (21%), self (16%), or public social services (12%). Participants were screened according to the criteria listed above and recruited during the 4-year period (1997–2001). Of the 302 families screened, 216 met the eligibility criteria…. The targeted families resided in Baltimore’s Westside Empowerment Zone (i.e., federally designated as an area of extreme poverty, unemployment, and general economic distress) and had at least one child between the ages of 5 and 11. Eligibility included (a) a concern by the referring person that at least 1 of 19 neglect subtypes (e.g., unsafe housing conditions, inadequate supervision, inadequate/delayed health care) was occurring at a low level but not at a level that Child Protective Services (CPS) would accept for investigation; (b) at least two additional risk factors for neglect related to the child (e.g., behavioral problem; physical, developmental, or learning disability; more than three children) or the caregiver/family (e.g., unemployment/ overemployment, mental health problem, drug or alcohol problem, domestic violence, homelessness); (c) no current CPS involvement; and (d) caregiver expressed willingness to participate in the FC program.^11^ | | Eligible schools were officially registered, had at least four classrooms and 120 students, were located in close proximity to other schools (i.e., ∼10 km or one hour walking), were in a secure zone at the time of school recruitment (e.g., no movement of armed groups), accessible by motorbike, and presumably not receiving support similar to OPEQ by other private, local, or international agencies.^12^ |  |
| CONSORT-SPI extension  When applicable, eligibility criteria for settings and those delivering the interventions | The following study inclusion criteria were used to control for any further variability in classroom characteristics across program type: (1) teachers with a bachelor’s degree or an associate’s degree and working towards a bachelor’s; (2) programs with moderate to high quality as measured by the NC (North Carolina) star-rating system (3–5 stars out of 5 stars total), (3) use of the Creative Curriculum (a state-approved MAF (More at Four Pre-Kindergarten Program) curriculum and the predominant curriculum used by MAF classrooms), (4) classroom enrollment of at least four Latino ELL (English Language Learner) children, but not to exceed 85% of total enrollment, and (5) use of English as the primary language of instruction.^13^ | |  |  |
| Item 4b.  Standard CONSORT description  Settings and locations where the data were collected | Our study site is St. Louis County, Missouri. We chose St. Louis County because it is part of a large metropolitan area with significant crime problems. In areas of the county patrolled by St Louis County Police Department (SLCPD), the 2012 violent crime rate was 244.6 per 100,000 while the property crime rate was 2063.8. Covering more than 500 square miles, with over 1 million residents, the county is the 34th largest in the U.S. and contains 17% of the state’s population, although the SLCPD provides primary police services to just over 400,000, including to more than 90 municipalities that contract for services. The SLCPD employs just over 800 sworn and 240 civilian personnel and is an internationally accredited, full-service department. Officers have fairly stable geographic assignments, ensuring some continuity across the treatment period.^14^ | |  |  |
| **Methods – Interventions**  Item 5.  Standard CONSORT description  The interventions for each group with sufficient details to allow replication, including how and when they were actually administered**^§^** | The integrated 12-Step facilitation (iTSF) intervention consisted of one 60–75-minute individual session, followed by eight weekly, 90-minute iTSF group sessions (n = 2-5 per group). Immediately prior to the fifth group therapy session, participants completed a second 30–50-minute individual ‘booster’ session. iTSF employed a ‘Socratic’ therapeutic questioning style to promote adolescent attention, verbal engagement and participation in discussion of topics, and therapists also used a variety of TSF strategies. At the end of each group, participants identified a sober activity goal for the week and reported on it at the beginning of the session the following week. Six sessions were based around a recovery-related topic, and two sessions invited members of 12-Step organizations to share their recovery story and experience. One therapist had a master’s degree in social work and was a certified Licensed Alcohol and Drug Counselor (LADC)-I with more than 5 years of experience. The other was in a clinical psychology doctoral training program, with several years of supervised clinical experience in substance use disorder (SUD) and mental health treatment. Both therapists had specific experience in cognitive behavioral therapy, 12-Step philosophy and principles and group-based interventions for individuals with SUD particularly with adolescents. Additionally, prior to beginning the study, therapists each attended five 12-Step meetings to familiarize themselves with the meeting format.^15^ | | In the parent and youth (PY) group, parents received the Familias: Preparando la Nueva Generación (FPNG) parenting curriculum, and youth received the youth-centered substance-use prevention program, keepin’ it REAL (kiR). In the youth group, youth received kiR, and parents did not receive any curriculum. In the control group, parents and youth received treatment-as-usual with respect to curricula offered at the schools.^16^ |  |
| Item 5a.  CONSORT-SPI extension  Extent to which interventions were actually delivered by providers and taken up by participants as planned | Treatment adherence and competence were monitored through weekly supervision and supervisor review of audio-recorded sessions. … In addition, four iTSF sessions (three group and one individual) were selected at random and rated by two independent doctoral level clinicians on three dimensions: adherence to protocol, skill level and frequency and extensiveness of skills used. On average, adherence was rated at 96.4%, and skill level as 6.4 – where 6 = ‘very good’ and 7 = ‘excellent’. Frequency and extensiveness was rated as 3.7 – where 3 = ‘adequately’ and 4 = ‘extensively’…. Of 59 participants who completed a baseline assessment, four never received any treatment, two of whom actively withdrew and two of whom were unable to be contacted; by the mid-treatment assessment, three more individuals withdrew consent; by end of treatment, two more participants withdrew consent. The study took place from July 2013 to October 2015.^15^ | |  |  |
| Item 5b.  CONSORT-SPI extension  Where other informational materials about delivering the intervention can be accessed | For more details, see: Kelly J. F., Yeterian J. D., Cristello J. C., Kaminer Y., Kahler C., Timko C. Developing and testing twelve-step facilitation for adolescents with substance use disorder: manual development and preliminary outcomes. Subst Abuse 2016; 10: 55–64.^15^ | |  |  |
| Item 5c.  CONSORT-SPI extension  When applicable, how intervention providers were assigned to each group | The two study therapists treated patients in both treatment conditions to avoid the problem of differential therapist effects. Individual motivational enhancement therapy (MET) sessions were divided equally across the two therapists and therapists co-led all groups in both conditions.^15^ | |  |  |
| **Methods – Outcomes**  Item 6a.  Standard CONSORT description  Completely defined pre-specified outcomes, including how and when they were assessed**^§^** | Irritable bowel syndrome symptom severity was measured using the Irritable Bowel Syndrome Severity Scoring System, which measures the severity of pain, distension, bowel dysfunction and quality of life/global well-being. Assessments were administered at four time-points: baseline (pretreatment), post-treatment (2 months) and at 3 and 6 months post-treatment. A decrease of 50 points on this scale has been identified as a clinically significant change in symptom severity.^17^ | | Viewsheds incorporate GIS methods to digitize the actual line-of-sight of CCTV cameras, which more accurately reflects camera coverage than traditional units of analysis, such as aggregate geographies (i.e., neighborhoods or police beats) and circular buffers drawn around camera sites. Researchers viewed the live feeds of all CCTV cameras in Newark and digitized the viewshed of each site within a GIS, … [which] created 75 separate CCTV schemes from the 146 individual viewsheds…. Catchment zones were created for each of the CCTV schemes [and served as the unit of measurement].^18^ |  |
| Item 6b.  Standard CONSORT description  Any changes to trial outcomes after the trial commenced, with reasons | We attempted to obtain collateral reports on the participants’ alcohol and drug use at the 12 month follow-up…. Because of the relatively low rate of apparent underreporting by participants relative to collaterals, and the small percentage of participants for whom collateral data were available, participant self-reports were used in the analyses of alcohol use. ^19^ | |  |  |
| **Methods – Sample Size**  Item 7a.  Standard CONSORT description  How sample size was determined**^§^** | Power calculations were performed using SAS PROC POWER for the primary aims of evaluating the equivalence of the UP and SDPs and evaluating the efficacy of the UP and SDPs relative to a benchmark WLC and were based on conventional target values of power = 0.80 and α = .05. With an allocation ratio of 2:1 for active treatment to WLC groups, results of the power calculations indicated that a sample size of 91 individuals per active treatment group provided adequate power for the analyses of both equivalence and superiority…. The equivalence margin of 0.75 Anxiety Disorders Interview Schedule clinical severity rating (ADIS CSR) units was selected based on available meta-analytic reviews of cognitive behavioral therapy outcome studies and recommendations for selecting a priori equivalence limits.^20^ | | For a medium effect size (d = 0.5, power = 0.80, α = .05), a sample size of 64 per group was needed to test for differences in means between two groups. For cluster RCTs, the sample size was adjusted according to the design effect (design effect = 1 + [cluster size −1] × ICC). As the design effect was 1.23 for parent and child outcomes and .277 for teacher reports, the required sample sizes were 158 and 433, respectively.^21^ |  |
| Item 7b.  Standard CONSORT description  When applicable, explanation of any interim analyses and stopping guidelines | An independent data monitoring committee reviewed unblinded data for safety after the first 1,000 women in the study had given birth. In response to a lower than anticipated attrition rate, we stopped recruitment when 1,748 had been randomly assigned.^22^ | |  |  |
| **Methods – Randomisation:** **Sequence Generation**  Item 8a.  Standard CONSORT description  Method used to generate the random allocation sequence | Random allocation was managed by the study statistician using computer-generated random numbers.^23^ | |  |  |
| Item 8b.  Standard CONSORT description  Type of randomisation; details of any restriction (such as blocking and block size)**^§^** | A blocked randomization scheme (blocks of 30) was used to yield balanced allocation of participants to treatment groups.^19^  Using a computerized adaptive minimization procedure, subjects were matched on suicide attempts or nonsuicidal self-injuries; psychiatric hospitalizations; history of suicide attempts and/or nonsuicidal self-injury; age; and a negative prognostic indicator of depression or a comorbid condition.^24^ | | School Report Card data were used to stratify schools into strata ranked on an index based on [individual] demographic variables; (b) characteristics of the student populations; and (c) indicators of student behavior and performance outcomes. Schools were matched on index score, resulting in 19 strata. Matched pairs were randomly selected from within strata, with one school of each pair randomly assigned to either intervention or control.^2^ |  |
| **Methods – Randomisation:** **Allocation Concealment Mechanism**  Item 9.  Standard CONSORT description  Mechanism used to implement the random allocation sequence, describing any steps taken to conceal the sequence until interventions were assigned**^§^** | The randomization list was transferred to a sequence of brown envelopes by writing the sequence of treatment names on the inside of the envelopes, which were then sealed. The sequence of envelopes was then ‘cut’ by taking approximately the first half of the envelopes and placing them at the end of the sequence so that no person involved in the trial would know the starting point of the randomization sequence and to preserve allocation concealment. The envelopes were then numbered.^25^ | | Schools agreeing to participate were stratified by percentage of children receiving free school meals (dichotomised at the median) and were randomly allocated within stratum to treatment arm. One researcher generated the allocation schedule using the Stats Direct computer program, the research unit co-coordinator allocated the schools to treatment arm blind to the identity of each school, and a second researcher enrolled schools.^26^ |  |
| **Methods – Randomisation:** **Implementation**  Item 10.  Standard CONSORT description  Who generated the random allocation sequence, who enrolled participants, and who assigned participants to interventions**^§^** | A study nurse telephoned a person at a randomization center who did not know the identities of the potential couples. The study nurse read the names from a list in order in which they had been assessed. Couples were randomly allocated by means of computer-generated random numbers. Every randomization result appeared in the program after the participants name was written and the person executing the randomization confirmed the process with her initials. This ensured that neither the study nurse, nor person doing the randomization could influence the result.^27,28^ | | General practices were the unit of randomization and determined the patients’ group status. GPs were randomised by coin toss after they gave their written informed consent. 136 GPs (15.9%) gave written informed consent to participate and agreed to adhere to the DelpHi trial protocol…. GPs assessed the eligibility of patients (≥70 years, living at home) and systematically screened patients who met the inclusion criteria…. All persons eligible for the study will be screened for cognitive impairment. People who met the inclusion criteria and provided their written informed consent to participate were included.^29,30^ |  |
| **Methods – Awareness of Assignment**  Item 11a.  Standard CONSORT description  Who was aware of intervention assignment after allocation (for example, participants, providers, those assessing outcomes), and how any masking was done | Although participants and clinicians delivering the treatment could not be blinded to treatment assignment, assessors and clinicians conducting outcome assessments were blinded. In addition, participants were instructed at their follow-up assessment interviews not to reveal their treatment assignment.^31^ | |  |  |
| Item 11b.  Standard CONSORT description  If relevant, description of the similarity of interventions | The attention control condition was led by the same interventionists who led the hypnosis intervention sessions. However, the interventionists did not lead the attention control patients in imagery, relaxation, or even simple discussion. Rather the interventionists allowed patients to direct the flow of the conversation and provided supportive and empathic comments according to standardized procedures.^32^ | |  |  |
| **Methods – Analytical Methods**  Item 12a.  Standard CONSORT description  Statistical methods used to compare group outcomes**^§^** | Linear random-effects models (hierarchical regression models) were implemented with random intercepts and slopes. These models estimate main effects for change from baseline to each assessment at 6, 12, and 18 months, main effect for the treatment, and interactions between the visit and treatment indicator variables. For each of the primary and secondary outcomes, separate intent-to-treat tests and estimates (with 95% CIs) of randomized group contrasts at 6, 12, and 18 months were obtained from the estimates of the respective time × treatment interactions. Potential confounding variables were evaluated by assessing whether baseline factors imbalanced between the treatment groups were related to outcome. Age was found to be a confounding variable and was controlled in all analyses.^33^ | Because schools were matched into pairs prior to randomization, the data presented here are nested: Children are nested in schools, and schools are nested in their matched pairs. To accommodate these design features, we calculated estimates of intervention impact on change in the primary child outcomes from preintervention baseline (fall 2004, Wave 1) to the first follow-up (spring 2005, Wave 2) using a series of two-level hierarchical linear models with random effects in HLM 6.02. In these models, Level 1 (child) included the preintervention baseline of the dependent variable and the child-level baseline covariates, and Level 2 (school) included a dummy variable indicating intervention condition as well as eight dummy variables representing the school pair matches.^34^ | |  |
| CONSORT-SPI extension  How missing data were handled, with details of any imputation method | For continuous outcomes, we used the multiple imputation procedure from SAS; because the data were non-monotonically missing, we used the Markov Chain Monte Carlo procedure. We used all available data regarding demographic status, psychosocial variables, unprotected anal intercourse, and transmission risk partners to impute missing values on risk outcomes. Missing data correction for binary measures used the previous wave value.^5^ |  | |  |
| Item 12b.  Standard CONSORT description  Methods for additional analyses, such as subgroup analyses, adjusted analyses, and process evaluations | We examined effect moderators using multiple regression. In Step 1, baseline conduct problem score was entered, followed by intervention status and moderator variable. In Step 2, the interaction term (Potential Moderator x Intervention Status) was introduced. We examined mediators by assessing associations between change in putative mediator, change in outcome, and intervention status; conducting hierarchical multiple regressions; and assessing significance using the Sobel test.^35^  The process evaluation was based on an inductive thematic analysis. Recordings were transcribed, coded, and analyzed by two researchers. Each researcher drew on the other to assess rater reliability and interpretation. Each session observed was reported separately first using a grounded theory approach with no observational protocol. Themes emerged through considering sessions and of archival materials.^36^ | |  |  |
| **Results – Participant Flow**  Item 13a.  Standard CONSORT description  For each group, the numbers randomly assigned, receiving the intended intervention, and analysed for the outcomes**^§^**  CONSORT-SPI extension  Where possible, the number approached, screened, and eligible prior to random assignment, with reasons for non-enrolment | See Appendix Figure 1. | | See Appendix Figure 1. |  |
| Item 13b.  Standard CONSORT description  For each group, losses and exclusions after randomisation, together with reasons**^§^** | Two of the 502 BMI (brief motivational intervention) participants were administratively dropped from the study when it was discovered after randomization that they began working at the university survey research center collecting data for the study.^37^ | |  |  |
| **Results – Recruitment**  Item 14a.  Standard CONSORT description  Dates defining the periods of recruitment and follow-up | Recruitment occurred from April 2006 to January 2008 using radio, web-based, and newspaper advertisements for a smoking cessation intervention consisting of group therapy plus nicotine patch…. Smoking was assessed at 1 week-, 4 weeks- (end of behavioral treatment), 16 weeks-, and 26 weeks-post assigned quit date.^38^ | |  |  |
| Item 14b.  Standard CONSORT description  Why the trial ended or was stopped | Recruitment was stopped once the study sample size was achieved because of study time lines and budget constraints, and the study was stopped once the 1-year follow-up assessments were completed.^39^ | |  |  |
| **Results – Baseline Data**  Item 15.  Standard CONSORT description  A table showing baseline characteristics for each group**^§^**  CONSORT-SPI extension  Include socioeconomic variables where applicable | See Table 1.^40^ | | See Tables 1 and 2.^41^ |  |
| **Results – Numbers Analysed**  Item 16.  Standard CONSORT description  For each group, number included in each analysis and whether the analysis was by original assigned groups**^§^** | See Table 6 for number of participants for each analysis. The primary analyses used all available follow-up data and compared participants in their randomized groups, irrespective of the intervention they received. The sensitivity of the primary analyses was assessed including baseline school attendance, using a per protocol analysis (excluding three participants in the psycho-education group, two of whom did not fulfil criteria for CFS (chronic fatigue syndrome) and one who received 13 sessions of CBT (cognitive behavioural therapy)) and multiple imputation as an alternative method for handling missing data.^25^ | | See Table 6. Importantly for our analyses, allocation was not associated with attrition. Results from complete case analyses (CCA; not tabled) were also carried out and did not differ markedly from those reported here. All models were carried out on the intent-to-treat basis and estimated controlling for student sex and baseline values of the evaluated outcome.^42^ |  |
| **Results – Outcomes and Estimation**  Item 17a.  Standard CONSORT description  For each outcome, results for each group, and the estimated effect size and its precision (such as 95% confidence interval)**^§^** | See Table 2 for adjusted and unadjusted summary results for each study group and the estimated effect size on continuous and dichotomous outcomes.^25^ | | See Table 2 for unadjusted summary results for each study group and the estimated effect size for each outcome. The average cluster size (number of participants in each preschool) was eight and the intracluster correlation (ρ) was 0.07. The design effect was thus 1.49.^43^ |  |
| CONSORT-SPI extension  Indicate availability of trial data | Data Availability: All relevant data are within the paper and its Supporting Information files.^44^ | |  |  |
| Item 17b.  Standard CONSORT description  For binary outcomes, presentation of both absolute and relative effect sizes is recommended | Based on observed data, 24.3% (N=27/111) of the patients in the CBT (cognitive behavioural therapy) condition and 21.3% (N=26/122) in the psycho- dynamic therapy condition met the remission criterion at the posttreatment assessment…. At the posttreatment assessment, the odds ratio was 0.82 (95% CI=0.45–1.50), indicating that remission rates did not differ significantly.^45^ | |  |  |
| **Results – Ancillary Analyses**  Item 18.  Standard CONSORT description  Results of any other analyses performed, including subgroup analyses, adjusted analyses, and process evaluations, distinguishing pre-specified from exploratory | There were no significant moderator effects for single parenthood, very low income, teen parenthood, and baseline level of observed child deviant behavior. Child gender, depression, and age were significant moderators, interacting with intervention status to predict conduct problem outcome. The intervention produced better conduct problems for boys, children of more depressed mothers, and younger children. Mediator analyses found change in positive parenting skill predicted change in conduct problems. See Tables 3-5.^35^  There was an openness and willingness from police officers to talk about their work in an accessible, engaging way. Young people were generally very well behaved and engaged in the sessions. Some facilitators were adept at holding information generated by the group and returning to it at relevant later points. The best facilitators did so in neutral, nonjudgmental ways. However, balancing the quantity of material with the quality of interaction was the biggest practical challenge observed. Supporting materials were difficult to implement systematically or consistently.^36^ | |  |  |
| **Results – Harms**  Item 19.  Standard CONSORT description  All-important harms or unintended effects in each group (for specific guidance see CONSORT for harms) | Regarding major incidents, two suicide attempts, two accidental deaths related to psychotic symptoms, a serious fight where the patients sustained serious injuries and three patients initiating substance abuse were recorded in the control group. In the family intervention group minor incidents were detected such as starting sexual relationships with the risk of HIV infection, vagrancy, bouts of alcohol consumption, and aggressivity.^46^ | |  |  |
| **Discussion – Limitations**  Item 20.  Standard CONSORT description  Trial limitations, addressing sources of potential bias, imprecision, and, if relevant, multiplicity of analyses | As described previously, statistical conclusion validity in the argumentation analysis was limited by not having a specific argumentation pretest covariate in the model, and instead a knowledge/reasoning pretest value for each student was used because a correlation was expected. Our claims about retention are similarly tempered (no argumentation pretest or no knowledge/reasoning retention measure). Other limitations of this study include the small sample size (58 students) and the short length of the intervention (10 hours of instruction, 4 hours of testing), yet the fact that we found significant and consistent differences despite these limitations speaks to the strength of the effect. Despite the teacher in this study having many years experience teaching both traditional and inquiry-based materials, he is undoubtedly more of an advocate of an inquiry-based approach. However, we believe the benefits of controlling variables by having the same teacher in both sections outweighed the potential bias created by a teacher being more comfortable in one approach than the other, and findings such as the comparable levels of student engagement shown in Table 4 suggest that the treatments were not strongly teacher-biased.^47^ | |  |  |
| **Discussion – Limitations**  Item 21.  Standard CONSORT description  Generalisability (external validity, applicability) of the trial findings**^§^** | Although the ethnic makeup of the sample roughly approximated that of the county in which our research center was situated (Middlesex County, Massachusetts), the sample included fewer African Americans, more biracial children, and participants with higher parental education and socioeconomic status than the general Middlesex County community. Further studies are needed to examine the protocol’s efficacy in samples with greater socioeconomic and ethnic diversity, as well its effectiveness in community mental health settings. In addition, our criteria allowed for the exclusion of children judged too uncooperative or distractible to take part in the treatment (two children) or children deemed too clinically severe to wait 6 months to receive treatment, based on severe mood disorder, severe social isolation, severe impairment in school function or attendance, or severe OCD (obsessive compulsive disorder) (a total of seven children). These criteria generally excluded children who clinically would not be administered CBT (cognitive behavioral therapy) for anxiety disorders as their first treatment (i.e., they might be offered such treatment after their other symptoms were addressed). Therefore, study results can be generalized only to children whose anxiety disorders are not so severe as to cause school refusal or severe social isolation. In other regards, however, the sample appeared representative of clinical samples, with high comorbidity of anxiety disorders and with 69% in the borderline or clinical range on the CBCL (child behavior checklist) Internalizing scale. In addition, our extensive intake assessment battery, which required a total of four parent and/or child visits prior to randomization, deterred as many as 1 in 4 potential participants and may have selected for families who were especially motivated to take part in treatment.^31^ | | In contrast to the lack of change shown in adolescents receiving creative workshops in Uganda, this intervention [in Indonesia], which includes structured creative activities as well as trauma-focused activities, did show effects on psychosocial well-being. It could therefore be considered a preliminary argument that increased structured interventions, which include trauma-focused activities, more effectively target PTSD symptoms. Corroboration of this argument can be found in the high-effect sizes of group CBT implemented in violence-affected schools in Los Angeles. However, previously mentioned qualitative research has shown the importance of addressing wider social problems caused by war, rather than purely focusing on PTSD complaints. In addition, specialized mental health professionals to implement CBT are usually unavailable in low-income settings. To resolve this tension, we propose that in complex emergencies, interventionists use a public health framework to tailor interventions to an appropriate population and referral level, based on investigated local needs, severity of complaints, available resources, and feasible and cost-effective interventions, while recognizing the importance of the social-ecological context. On the basis of these findings, the classroom-based intervention then qualifies as an appropriate intervention to target larger groups of children (especially girls) at risk, when stress-related symptoms are relevant.^48^ |  |
| **Discussion – Interpretation**  Item 22.  Standard CONSORT description  Interpretation consistent with results, balancing benefits and harms, and considering other relevant evidence | The results from this trial differed from previous CBT (cognitive behavioural therapy) trials in two key areas. Only one patient (3%) did not complete the treatment. Previous IBS (irritable bowel syndrome) studies suggest that drop-out rates from CBT can be as high 40%. This may be because traditional CBT requires a substantial time commitment from patients. The most common reasons for dropping out are being unable to take time off work or childcare commitments. Having fewer sessions and sessions on the telephone may make the therapy more widely available. In addition, presenting treatment as self-management of a chronic condition rather than as a psychological therapy may be more acceptable to IBS patients. The treatment effects for symptom severity in this study are larger than those reported in many other CBT trials. This may be because of differences in the patient cohorts. As our study did not rely on GP (general practitioner) referral we may have accessed a cohort that seldom gets offered therapeutic intervention or perhaps even gets diagnosed. This is important, as our results suggest that treatment effects may be greater if patients are less disabled by their symptoms and less depressed. There is certainly evidence that depression in IBS is related to poorer treatment outcome. This study indicates that early intervention and diagnosis may not only make treatment more effective but also prevent the illness becoming more chronic and refractory to treatment.^17^ | |  |  |
| **Important Information** **- Registration**  Item 23.  Standard CONSORT description  Registration number and name of trial registry | We registered the study with the American Economic Association (AEARCTR-0000742).^49^ | |  |  |
| **Important Information** **- Protocol**  Item 24.  Standard CONSORT description  Where the full trial protocol can be accessed, if available | The full trial protocol is available in the Supplement:  https://jamanetwork.com/data/Journals/PSYCH/935708/YOI160049supp1_prod.pdf.^50^ | |  |  |
| **Important Information** **–**  **Declaration of Interests**  Item 25.  Standard CONSORT description  Sources of funding and other support, role of funders | This work was supported by a research grant from the National Institute on Drug Abuse (R01 DA015183-05) with cofunding from the National Cancer Institute, the National Institute of Child Health and Human Development, the National Institute of Mental Health, and the Center for Substance Abuse Prevention. The funders had no role in the design, conduct, analysis and reporting of the trial.^51^ | |  |  |
| CONSORT-SPI extension  Declaration of any other potential interests | The contributions of GP in reviewing the creative process instrument a few times are gratefully acknowledged. GP authored a few books such as Evidence based teaching—A practical approach (2006), Teaching today: A practical guide (2004) and How to be better at creativity (1996). He works for the Learning Skills Development Agency as a consultant on their Raising Quality and Achievement programme, assisting Action Research Development Projects in colleges, and assisting the Quality Improvement Team. He is a visiting examiner for the Institute of Education at London University. His experience includes physics teaching, managing teacher training, Inclusive Learning Facilitator, being a staff development officer, and managing college lesson observation.”^52^ | |  |  |
| **Important Information** **–**  **Stakeholder Involvement**  Item 26a.  CONSORT-SPI extension  Any involvement of the intervention developer in the design, conduct, analysis, and reporting of the trial | The evaluation was conducted by the same team of people that designed the intervention. In order to promote objectivity of the evaluation we submitted the trial protocol for publication before the study began and submitted statistical analysis plans with prespecified primary outcomes before data were inspected, and these plans were scrutinized by an independent data-monitoring committee. As with all new projects, it is likely that the intervention was managed and implemented with greater expertise and enthusiasm by the project team than could be expected in subsequent iterations and scaling up of the intervention.^53^ | |  |  |
| Item 26b.  CONSORT-SPI extension  Other stakeholder involvement in trial design, conduct, and/or analyses | The SKCHH [Seattle–King County Healthy Homes] project was designed as a community-based participatory research project with overall sponsorship by Seattle Partners for Healthy Communities, an Urban Research Center funded by the U.S. Centers for Disease Control and Prevention. Seattle Partners is a multidisciplinary partnership of community agencies, community activists, public health professionals, academics, and health providers that supports community-based participatory research addressing social determinants of health…. Both the Seattle Partners Board and the steering committee sought to assure that the project benefited all participants. This led to the staggered intervention design with low- and high-intensity groups. This design assured that low-intensity group participants initially received some immediate benefit (including interventions known to be useful, such as bedding encasements) while ultimately receiving all the benefits accorded the high-intensity group. While this design may have reduced the study’s power to demonstrate an effect of the high- intensity intervention relative to a “pure” control group receiving no intervention, we felt such a design was not ethical.”^54^ | |  |  |
| Item 26c.  CONSORT-SPI extension  Incentives offered as part of the trial | As part of an incentive package to each participating school, all teachers received free OCR program materials and professional development supports throughout the year. Control schools from five districts received a core math program (Everyday Mathematics) and related professional development at no charge, whereas two districts received a cash incentive of $5,000 per school in each year of the study. In this sense, the treatment and control schools received a similar amount of resources, with treatment schools receiving additional supports for their literacy programs and control schools receiving additional supports for their core math or other educational programs. Additionally, the study provided incentives to participants. Teachers received $15 for completing a survey in the fall and spring of each year. In addition, teachers received up to $45 for completing an interview and for allowing researchers to observe their classrooms up to three times per year. A staff person from each school volunteered as a school liaison and received a $500 stipend per year for coordinating research activities at each school.^55^ | |  |  |

**References**

1. Rosen D, Engel R, McCall J, Greenhouse J. Using problem-solving therapy to reduce depressive symptom severity among older adult methadone clients: A randomized clinical trial. *Research on Social Work Practice.* 2017.

2. Snyder F, Flay B, Vuchinich S, et al. Impact of a social-emotional and character development program on school-level indicators of academic achievement, absenteeism, and disciplinary outcomes: A matched-pair, cluster-randomized, controlled trial. *Journal of Research on Educational Effectiveness.* 2009;3(1):26-55.

3. Anderson T, Guajardo JF, Luthra R, Edwards KM. Effects of clinician-assisted emotional disclosure for sexual assault survivors: A pilot study. *Journal of Interpersonal Violence.* 2010;25:1113-1131.

4. Averdijk M, Zirk-Sadowski J, Ribeaud D, Eisner M. Long-term effects of two childhood psychosocial interventions on adolescent delinquency, substance use, and antisocial behavior: A cluster randomized controlled trial. *Journal of Experimental Criminology.* 2016;12(1):21-47.

5. McKirnan DJ, Tolou-Shams M, Courtenay-Quirk C. The Treatment Advocacy Program: A randomized controlled trial of a peer-led safer sex intervention for HIV-infected men who have sex with men. *Journal of Consulting and Clinical Psychology.* 2010;78(6):952–963.

6. Borman GD, Dowling NM, Schneck C. A multisite cluster randomized field trial of Open Court Reading. *Educational Evaluation and Policy Analysis.* 2008;30(4):389-407.

7. Green J, Pickles A, Pasco G, et al. Randomised trial of a parent‐mediated intervention for infants at high risk for autism: longitudinal outcomes to age 3 years. *Journal of Child Psychology and Psychiatry.* 2017;58(12):1330–1340.

8. Webb MS, de Ybarra DR, Baker EA, Reis IM, Carey MP. Cognitive-behavioral therapy to promote smoking cessation among African American smokers: A randomized clinical trial. *Journal of Consulting and Clinical Psychology.* 2010;78(1):24-33.

9. Buller DB, English DR, Buller MK, et al. Shade sails and passive recreation in public parks of Melbourne and Denver: a randomized intervention. *American Journal of Public Health.* 2017;107(12):1869-1875.

10. Chang HY, Quintana C, Krajcik JS. The impact of designing and evaluating molecular animations on how well middle school students understand the particulate nature of matter. *Science Education.* 2010;94(1):73-94.

11. Lindsey MA, Hayward A, DePanfilis D. Gender differences in behavioral outcomes among children at risk of neglect: Findings from a family-focused prevention intervention. *Research on Social Work Practice.* 2010;20(6):572-581.

12. Aber JL, Torrente C, Starkey L, et al. Impacts after one year of “Healing Classroom” on children's reading and math skills in DRC: Results from a cluster randomized trial. *Journal of Research on Educational Effectiveness.* 2017;10(3):507-529.

13. Buysse V, Castro DC, Peisner-Feinberg E. Effects of a professional development program on classroom practices and outcomes for Latino dual language learners. *Early Childhood Research Quarterly.* 2010;25:194-207.

14. Kochel TR, Weisburd D. Assessing community consequences of implementing hot spots policing in residential areas: Findings from a randomized field trial. *Journal of Experimental Criminology.* 2017;13(2):143-170.

15. Kelly JF, Kaminer Y, Kahler CW, et al. A pilot randomized clinical trial testing integrated 12-Step facilitation (iTSF) treatment for adolescent substance use disorder. *Addiction.* 2017;112:2155–2166.

16. Williams LR, Ayers S, Baldwin A, Marsiglia FF. Delaying youth substance-use initiation: A cluster randomized controlled trial of complementary youth and parenting interventions. *Journal of the Society for Social Work and Research.* 2016;7(1):177-200.

17. Moss-Morris R, McAlpine L, Didsbury LP, Spence MJ. A randomized controlled trial of a cognitive behavioural therapy-based self-management intervention for irritable bowel syndrome in primary care. *Psychological Medicine.* 2010;40:85–94.

18. Piza EL, Caplan JM, Kennedy LW, Gilchrist AM. The effects of merging proactive CCTV monitoring with directed police patrol: A randomized controlled trial. *Journal of Experimental Criminology.* 2015;11(1):43-69.

19. McKay JR, Van Horn DHA, Oslin DW, et al. A randomized trial of extended telephone-based continuing care for alcohol dependence: Within-treatment substance use outcomes. *Journal of Consulting and Clinical Psychology.* 2010;78(6):912-923.

20. Barlow DH, Farchione TJ, Bullis JR, et al. The unified protocol for transdiagnostic treatment of emotional disorders compared with diagnosis-specific protocols for anxiety disorders: A randomized clinical trial. *JAMA Psychiatry.* 2017;74(9):875-884.

21. Leung C, Tsang S, Kwan HW. Efficacy of a universal parent training program (HOPE-20): Cluster randomized controlled trial. *Research on Social Work Practice.* 2015;27(5):523-537.

22. Tracy SK, Hartz DL, Tracy MB, et al. Caseload midwifery care versus standard maternity care for women of any risk: M@NGO, a randomised controlled trial. *Lancet.* 2013;382(9906):1723-1732.

23. Gleeson JFM, Cotton SM, Alvarez-Jimenez M, et al. Family outcomes from a randomized control trial of relapse prevention therapy in first-episode psychosis. *Journal of Clinical Psychiatry.* 2010;71(4):475-483.

24. Linehan MM, Comtois KA, Murray AM, et al. Two-year randomized controlled trial and follow-up of dialectical behavior therapy vs therapy by experts for suicidal behaviors and borderline personality disorder. *Archives of General Psychiatry.* 2006;63(7):757-766.

25. Chalder T, Deary V, Husain K, Walwyn R. Family-focused cognitive behaviour therapy versus psycho-education for chronic fatigue syndrome in 11- to 18-year-olds: A randomized controlled treatment trial. *Psychological Medicine.* 2010;40:1269–1279.

26. Mulvaney CA, Kendrick D, Watson MC, Coupland CA. Increasing child pedestrian and cyclist visibility: Cluster randomised controlled trial. *Journal of Epidemiology & Community Health.* 2006;60(4):311-315.

27. Laakkonen ML, Hölttä EH, Savikko N, Strandberg TE, Suominen M, Pitkälä KH. Psychosocial group intervention to enhance self-management skills of people with dementia and their caregivers: Study protocol for a randomized controlled trial. *Trials.* 2012;13(1):133.

28. Laakkonen ML, Savikko N, Hölttä E, et al. Self-management groups for people with dementia and their spousal caregivers. A randomized, controlled trial. Baseline findings and feasibility. *European Geriatric Medicine.* 2013;4(6):389-393.

29. Thyrian JR, Fiß T, Dreier A, et al. Life-and person-centred help in Mecklenburg-Western Pomerania, Germany (DelpHi): study protocol for a randomised controlled trial. *Trials.* 2012;13(1):56.

30. Thyrian JR, Hertel J, Wucherer D, et al. Effectiveness and safety of dementia care management in primary care: A randomized clinical trial. *JAMA Psychiatry.* 2017;74(10):996-1004.

31. Hirshfeld-Becker DR, Masek B, Henin A, et al. Cognitive behavioral therapy for 4- to 7-year-old children with anxiety disorders: A randomized clinical trial. *Journal of Consulting and Clinical Psychology.* 2010;78(4):498-510.

32. Montgomery GH, Hallquist MN, Schnur JB, David D, Silverstein JH, Bovbjerg DH. Mediators of a brief hypnosis intervention to control side effects in breast surgery patients: Response expectancies and emotional distress. *Journal of Consulting and Clinical Psychology.* 2010;78(1):80-88.

33. Grant PM, Huh GA, Perivoliotis D, Stolar NM, Beck AT. Randomized trial to evaluate the efficacy of cognitive therapy for low-functioning patients with schizophrenia. *Archives of General Psychiatry.* 2012;69(2):121-127.

34. Jones SM, Brown JL, Hoglund WL, Aber JL. A school-randomized clinical trial of an integrated social–emotional learning and literacy intervention: Impacts after 1 school year. *Journal of Consulting and Clinical Psychology.* 2010;78(6):829-842.

35. Gardner F, Hutchings J, Bywater T, Whitaker C. Who benefits and how does it work? Moderators and mediators of outcome in an effectiveness trial of a parenting intervention. *Journal of Clinical Child & Adolescent Psychology.* 2010;39(4):568-580.

36. Densley JA, Adler JR, Zhu L, Lambine M. Growing against gangs and violence: Findings from a process and outcome evaluation. *Psychology of Violence.* 2017;7(2):242.

37. Wood MD, Fairlie AM, Fernandez AC, et al. Brief motivational and parent interventions for college students: A randomized factorial study. *Journal of Consulting and Clinical Psychology.* 2010;78(3):349-361.

38. MacPherson L, Tull MT, Matusiewicz AK, et al. Randomized controlled trial of behavioral activation smoking cessation treatment for smokers with elevated depressive symptoms. *Journal of Consulting and Clinical Psychology.* 2010;78(1):55-61.

39. Mills M, Loney P, Jamieson E, et al. A primary care cardiovascular risk reduction clinic in Canada was more effective and no more expensive than usual on-demand primary care: A randomised controlled trial. *Health & Social Care in the Community.* 2010;18(1):30-40.

40. Manne S, Jacobsen PB, Ming ME, Winkel G, Dessureault S, Lessin SR. Tailored versus generic interventions for skin cancer risk reduction for family members of melanoma patients. *Health Psychology.* 2010;29(6):583-593.

41. Donnelly JE, Hillman CH, Greene JL, et al. Physical activity and academic achievement across the curriculum: Results from a 3-year cluster-randomized trial. *Preventive Medicine.* 2017;99:140-145.

42. Obsuth I, Sutherland A, Cope A, Pilbeam L, Murray AL, Eisner M. London Education and Inclusion Project (LEIP): Results from a cluster-randomized controlled trial of an intervention to reduce school exclusion and antisocial behavior. *Journal of Youth and Adolescence.* 2017;46(3):538-557.

43. Leung C, Tsang S, Heung K. The effectiveness of healthy start home visit program: cluster randomized controlled trial. *Research on Social Work Practice.* 2015;25(3):322-333.

44. Trinies V, Chard AN, Mateo T, Freeman MC. Effects of water provision and hydration on cognitive function among primary-school pupils in Zambia: A randomized trial. *PLoS One.* 2016;11(3):e0150071.

45. Driessen E, Van HL, Don FJ, et al. The efficacy of cognitive-behavioral therapy and psychodynamic therapy in the outpatient treatment of major depression: A randomized clinical trial. *American Journal of Psychiatry.* 2013;170:1041-1050.

46. Girón M, Fernández-Yañez A, Mañá-Alvarenga S, Molina-Habas A, Nolasco A, Gómez-Beneyto M. Efficacy and effectiveness of individual family intervention on social and clinical functioning and family burden in severe schizophrenia: A 2-year randomized controlled study. *Psychological Medicine.* 2010;40:73-84.

47. Wilson CD, Taylor JA, Kowalski SM, Carlson J. The relative effects and equity of inquiry‐based and commonplace science teaching on students' knowledge, reasoning, and argumentation. *Journal of Research in Science Teaching.* 2010;47(3):276-301.

48. Tol WA, Komproe IH, Susanty D, Jordans MJ, Macy RD, De Jong JT. School-based mental health intervention for children affected by political violence in Indonesia: A cluster randomized trial. *JAMA.* 2008;300(6):655-662.

49. Goesling B, Scott ME, Cook E. Impacts of an enhanced family health and sexuality module of the HealthTeacher middle school curriculum: A cluster randomized trial. *American Journal of Public Health.* 2016;106(S1):S125-S131.

50. Gibbons MBC, Gallop R, Thompson D, et al. Comparative effectiveness of cognitive therapy and dynamic psychotherapy for major depressive disorder in a community mental health setting: a randomized clinical noninferiority trial. *JAMA Psychiatry.* 2016;73(9):904-911.

51. Cho K, MacArthur C. Student revision with peer and expert reviewing. *Learning & Instruction.* 2010;20:328-338.

52. Leng EY, Ali WZbW, Mahmud Rb, Baki R. Computer games development experience and appreciative learning approach for creative process enhancement. *Computers & Education.* 2010;55:1131-1144.

53. Jukes MC, Turner EL, Dubeck MM, et al. Improving literacy instruction in Kenya through teacher professional development and text messages support: A cluster randomized trial. *Journal of Research on Educational Effectiveness.* 2017;10(3):449-481.

54. Krieger J, Takaro TK, Allen C, et al. The Seattle–King County Healthy Homes Project: Implementation of a comprehensive approach to improving indoor environmental quality for low-income children with asthma. *Environmental Health Perspectives.* 2002;110(Supplement 2):311-322.

55. Vaden-Kiernan M, Borman G, Caverly S, et al. Findings from a multiyear scale-up effectiveness trial of open court reading. *Journal of Research on Educational Effectiveness.* 2017.
