# Supplementary material for: CONSORT-SPI 2018 Explanation and Elaboration: guidance for reporting social and psychological intervention trials
Source: Trials. 2018 Jul 31;19:406. doi: 10.1186/s13063-018-2735-z (PMC6066913; doi:10.1186/s13063-018-2735-z)
Supplement: Supplementary file 3 — Table S3. Examples of abstracts adherent to the CONSORT-SPI Extension for Abstracts. (DOCX 73 kb) [file 13063_2018_2735_MOESM3_ESM.docx]

**Additional file 3: Table S3. Examples of abstracts adherent to the CONSORT-SPI Extension for Abstracts***

| **Example for a individually-randomised trial** | **Example for a cluster-randomised trial** |
| --- | --- |
| Title: Effectiveness of online collaborative care for treating mood and anxiety disorders in primary care: A randomized clinical trial.^1^  Importance: Collaborative care for depression and anxiety is superior to usual care from primary care physicians for these conditions; however, challenges limit its provision in routine practice and at scale.  Objective: To examine the effectiveness of combining an internet support group (ISG) with an online computerized cognitive behavioural therapy (CCBT) provided via a collaborative care program for treating depression and anxiety versus CCBT alone and whether providing CCBT in this manner is more effective than usual care. ISG aims to use advances in technology to overcome barriers to the effectiveness of CCBT.  Design: In this 3-arm randomized clinical trial with blinded outcome assessments, primary care physicians from 26 primary care practices in Pittsburgh, Pennsylvania that shared a common electronic medical record (EMR) referred 2884 patients aged 18 to 75 years in response to an electronic medical record prompt from August 2012 to September 2014. Eligible patients needed to have internet and email access; a score of 10 or greater on either the 7-Item Generalized Anxiety Disorder scale (GAD-7) or the 9-Item Patient Health Questionnaire (PHQ-9); and no alcohol dependence as determined by the Alcohol Use Disorders Identification Test, active suicidality, or other serious mental illness for which our interventions may be inappropriate. Overall, 704 patients (24.4%) met all eligibility criteria and were randomized to CCBT alone (n = 301), CCBT+ISG (n = 302), or usual care (n = 101). Intent-to-treat analyses were conducted November 2015 to January 2017.  Interventions: Six months of guided access to an 8-session CCBT program provided by care managers who informed primary care physicians of their patients’ progress and promoted patient engagement with our online programs. We employed college graduates with mental health research experience as care managers and assigned each exclusively to one intervention arm. We first prepared them in a basic understanding of mood and anxiety disorders, our pharmacotherapy algorithm, CCBT program, and tracking registry and later reinforced this training in our weekly case review sessions.  Measures: Mental health–related quality of life (12-Item Short-Form Health Survey Mental Health Composite Scale; primary outcome) and depression and anxiety symptoms (Patient-Reported Outcomes Measurement Information System; secondary outcomes) at 6-month follow-up, with treatment durability assessed 6 months later.  Results: Of the 704 randomized patients, 562 patients (79.8%) were female, and the mean (SD) age was 42.7 (14.3) years. A total of 604 patients (85.8%) completed our primary 6-month outcome assessment. At 6-month assessment, 254 of 301 patients (84.4%) receiving CCBT alone started the program (mean [SD] sessions completed, 5.4 [2.8]), and 228 of 302 patients (75.5%) in the CCBT+ISG cohort logged into the ISG at least once, of whom 141 (61.8%) provided 1 or more comments or posts (mean, 10.5; median [range], 3 [1-306]). At 6-month follow-up, patients in the CCBT+ISG (n = 302) and CCBT alone (n = 301) arms reported similar improvements on our primary outcome measure (SF-12 MCS: ES, 0.02; 95% CI, −0.17 to 0.13) However, compared with patients receiving usual care (n = 101), patients in the CCBT alone cohort reported significant 6-month effect size improvements in mood (effect size, 0.31; 95% CI, 0.09-0.53) and anxiety (effect size, 0.26; 95% CI, 0.05-0.48) that persisted 6 months later, and completing more CCBT sessions produced greater effect size improvements in mental health–related quality of life and symptoms. No adverse events or side effects were reported.  Conclusions: While providing moderated access to an ISG provided no additional benefit over guided CCBT at improving mental health–related quality of life, mood, and anxiety symptoms, guided CCBT alone is more effective than usual care for these conditions.  Trial Registration: NCT01482806 in clinicaltrials.gov  Funding: This work was supported by grant R01 MH093501 from the National Institute of Mental Health. | Title: Effects of Smokefree Class Competition 1 year after the end of intervention: A cluster randomised controlled trial.^2^  Importance: The Smokefree Class Competition (SFC), a school-based smoking prevention intervention, is widely disseminated in Europe.  Objective: Effects of the intervention on individual students’ smoking, initiation, and progression of smoking were investigated. SFC aims to correct the overestimation of smoking frequency in adolescents by demonstrating that the class, an important reference group for students, is smoke-free.  Design: Cluster randomised controlled trial. Saxony-Anhalt, Germany was chosen as the study region because SFC was rarely implemented there before the school year 2006–2007. ‘Gymnasium’ and ‘Sekundarschule’ secondary schools were invited to participate (Gymnasium N=71, Sekundarschule N=141). After applying the school exclusion criteria (a closure of the school was foreseen in the following 2 years, the school was already engaged in a tobacco control programme, or classes of the school had participated in SFC before), 84 schools (including its classes and students) were randomly assigned to intervention (53 schools, 137 classes, 2,629 students) or control condition (34 schools, 86 classes, 1,825 students).  Interventions: SFC classes commit themselves to be smoke-free and self-monitor their smoking status. Classes that remain smoke-free for 6 months can win prizes. The classes in control condition received ‘usual curriculum.’  Measures: The primary outcome was student self-reported (i.e., unblinded) current smoking 12 months after baseline. Effects of participation in the programme were analysed by multilevel models controlling for confounding variables.  Results: Overall, 68 classes (1,175 students) agreed to participate in SFC. There was no significant difference between students who were assigned to and participated in SFC (9 classes, 237 students) and students who did not participate in SFC or were in the control condition (11 classes, 214 students) on current smoking (adjusted hazard ratio, 0.06, 95% CI, -0.06 to 0.18). However, intervention students smoking occasionally at baseline smoked less frequently than students taking not part in the intervention at 7 and 12 months after baseline. Persistent beneficial programme effects were also found for lifetime smoking: intervention students were less likely to progress from experimental to established use. No adverse events or side effects were reported.  Conclusion: Data suggest that Smokefree Class Competition reduces the probability of progressing from occasional and experimental stages of smoking to more established forms of use.  Trial registration: ISRCTN27091233 in Current Control Trial Register.  Funding: This study was funded by German Cancer Aid. The implementation of the Smokefree Class Competition in Germany was funded by German Cancer Aid, European Commission, Federal Centre for Health Education, German Heart Foundation, AOK-Bundesverband, German Lung Foundation and BKK-Landesverband Ost in the school year 2006/2007 and 2007/2008. |

CONSORT = Consolidated Standards of Reporting Trials; SPI = social and psychological intervention.

*Original abstracts were modified to adhere to the CONSORT-SPI Extension for Abstracts.

**References**

1. Rollman BL, Belnap BH, Abebe KZ, et al. Effectiveness of online collaborative care for treating mood and anxiety disorders in primary care: a randomized clinical trial. *JAMA Psychiatry.* 2017.

2. Isensee B, Morgenstern M, Stoolmiller M, Maruska K, Sargent JD, Hanewinkel R. Effects of Smokefree Class Competition 1 year after the end of intervention: a cluster randomised controlled trial. *Journal of Epidemiology & Community Health.* 2012;66(4):334-341.
