# Supplementary material for: CONSORT-SPI 2018 Explanation and Elaboration: guidance for reporting social and psychological intervention trials
Source: Trials. 2018 Jul 31;19:406. doi: 10.1186/s13063-018-2735-z (PMC6066913; doi:10.1186/s13063-018-2735-z)
Supplement: Supplementary file 4 — Figure S1. Example of a participant flow diagram. (DOCX 111 kb) [file 13063_2018_2735_MOESM4_ESM.docx]

**Additional file 4: Figure S1. Example of a participant flow diagram^49^**

## Approach

## Allocation

Excluded (n = 2 schools)

♦  Low consent (n = 2 schools)

Approached (n = 19 schools)

Screened/assessed for eligibility

(n = 17 schools, 2,149 students)

Excluded (n = 615 students)

♦  No consent for data collection (n = 615 students)

Randomised

(n = 17 schools, 1,534 students)

## Enrollment

- Allocated to intervention (n = 779 students)

♦ Daily attendance rates (94-98%)

- Schools (n = 9)

♦ Number of students per schools

(median = 94, range 39 to 112)

- Allocated to intervention (n = 775 students)

♦ Daily attendance rates (Not reported)

- Schools (n = 8)

♦ Number of students per schools

(median = 83, range 36 to 163)

## Follow-Up

Lost to follow-up

♦ Schools (n = 0)

♦ Student who did not complete follow-up at

9 months (n = 39)

♦ Student who did not complete follow-up at

15 months (n = 99)

Discontinued intervention (give reasons) (n= )

Lost to follow-up

♦ Schools (n = 0)

♦ Student who did not complete follow-up at

9 months (n = 51)

♦ Student who did not complete follow-up at

15 months (n = 51)

Discontinued intervention (give reasons) (n= )

## Analysis

Analysed (n = 7 schools)

♦ Students at 9 months (n = 625)

♦ Students at 15 months (n = 595)

Excluded from analysis (n = 2)

♦ Outlier on rates of sexual activity (n = 1)

♦ Randomised without a match (n = 1)

Analysed (n = 7 schools)

♦ Students at 9 months (n = 651)

♦ Students at 15 months (n = 594)

Excluded from analysis (n = 1)

♦ Randomised with outlier school (n = 1)
